# Deep spatial sequencing revealing differential immune responses in human hepatocellular carcinoma

**DOI:** 10.3389/fcell.2025.1600129

**Published:** 2025-06-03

**Authors:** Yan-Ping Yu, Caroline Obert, Bao-Guo Ren, Marielle Krivit, Kyle Metcalfe, Jia-Jun Liu, Tuval Ben-Yehezkel, Silvia Liu, Jian-Hua Luo

**Affiliations:** ^1^ Departments of Pathology, University of Pittsburgh School of Medicine, Pittsburgh, PA, United States; ^2^ Element Biosciences Inc., San Diego, CA, United States; ^3^ Department of Pharmacology and Chemical Biology, University of Pittsburgh School of Medicine, Pittsburgh, PA, United States; ^4^ Institute of Organ Pathobiology and Therapeutics, University of Pittsburgh School of Medicine, Pittsburgh, PA, United States

**Keywords:** ultra-depth spatial sequencing, HCC, cancer microenvironment, cell-cell interaction, immune responses

## Abstract

Hepatocellular carcinoma (HCC) is one of the most lethal cancers for humans. HCC is highly heterogeneous. In this study, we performed ultra-depth (∼1 million reads per spot) sequencing of 6,320 spatial transcriptomes on a case of HCC. Sixteen distinct spatial expression clusters were identified. Each of these clusters was spatially contiguous and had distinct gene expression patterns. In contrast, benign liver tissues showed minimal heterogeneity in terms of gene expression. Numerous immune cell-enriched spots were identified in both HCC and benign liver regions. Cells adjacent to these immune cell-enriched spots showed significant alterations in their gene expression patterns. Interestingly, the responses of HCC cells to the nearby immune cells were significantly more intense and broader, while the responses of benign liver cells to immune cells were somewhat narrow and muted, suggesting an innate difference in immune cell activities towards HCC cells in comparison with benign liver cells. However, cell-cell interaction analyses showed significant immune evasion by HCC cancer cells. When standard-depth sequencing was performed, significant numbers of genes and pathways that were associated with these changes disappeared. Qualitative differences in some pathways were also found. These results suggest that deep spatial sequencing may help to uncover previously unidentified mechanisms of liver cancer development.

## Introduction

Liver cancer is one of the most lethal malignancies for humans and causes over 700,000 deaths worldwide annually (Jemal et al., 2012; Jemal et al., 2012; McGuire, 2016). Hepatocellular carcinoma (HCC) is the most common type of liver cancer and accounts for 90% of all liver cancers (Siegel et al., 2022; Giaquinto et al., 2022). Large numbers of genomic and gene expression abnormalities have been discovered in HCC (Khemlina et al., 2017; Yu et al., 2024; Liu et al., 2024a; Kader et al., 2024a; Kader et al., 2024b; Zuo et al., 2022; Liu et al., 2022; Luo et al., 2021; Yu et al., 2019; He et al., 2017; Chen et al., 2017a; Chen et al., 2017b; Nalesnik et al., 2012; Luo et al., 2006; Yu et al., 2013). HCC has high levels of heterogeneity. These heterogeneities may result from the underlying variation of genomic alterations. However, the location-based heterogeneity of HCC has rarely been analyzed at the genetic level.

In recent years, spatial genetic analysis has been rapidly developed. Cancer microenvironment has been shown to have significant variations in different cancer locations (Li et al., 2022). Studies utilizing spatial genetic analysis revealed spatial relationships of cell-cell interaction and the impact of genetic alterations of a cell on its adjacent tissue microenvironment (Arora et al., 2023). Due to the significant genetic heterogeneity in an HCC sample, the tumor microenvironment may differ from region to region. In this report, we performed a 10x Genomics Visium Spatial transcriptome analysis on a case of HCC samples. Significant variation of gene expression patterns was found in different regions of the cancer samples.

## Materials and methods

### Tissue samples and CytAssist workflow

A case of HCC sample with moderate differentiation and a history of alcohol abuse and Hepatitis B infection was obtained from archived tissue slide storage. The case was fully anonymized. The tissue procurement protocols were approved by the Institutional Review Board of University of Pittsburgh. All procedure and protocols were carried out in accordance with the guidelines by the Institutional Review Board of University of Pittsburgh. The cancer and benign liver regions were identified by a board-certified pathologist. The slides were deparaffinized, Hematoxylin/Eosin stained, and underwent Visium cassette assembly, probe hybridization/ligation, tissue transfer to Visium slide, DNA isolation, clean-up, and ligation probe amplification based on the manufacturer’s manual. Standard sequencing was performed on Illumina NextSeq 550 Dx platform, while ultra-depth sequencing was performed on the Element Biosciences AVITI platform. The sequencing procedures followed the manufacturers’ recommendations (Liu et al., 2022; Luo et al., 2021; Yu et al., 2013; Liu et al., 2024b; Yu et al., 2014; Liu et al., 2024c; Luo et al., 2015).

### Statistical analysis for spatial transcriptomics data

Spatial transcriptomics was measured by the Visium CytAssist platform. The H&E staining file was imported to the Loupe Browser (10x Genomics) for spatial alignment. Then, the alignment file and the raw sequencing FASTQ files were processed by Space Ranger (10x Genomics) to align to the human reference genome hg38. After pre-processing, the feature by cell count matrix and the imaging files were analyzed by the R Seurat package (Hao et al., 2024). To integrate two slides, top 3,000 high-variable genes to integrate the two libraries using SCT transformation. Principal component analysis followed by the Uniform Manifold Approximation and Projection (UMAP) was applied for dimension reduction and visualization. Spatial spots were clustered based on the gene expression profiles. Spatial transcriptomics data were visualized with UMAP and spatial feature plot provided by the Seurat package.

To check the immune cells and their tissue microenvironments, spots with high immune expression were identified and further analyzed. Kupffer cells were identified by CD68, CD163, LYZ, C1QA, AIF1; T cells were identified by CD3D, CD2, IL7R, TRBC2, CD69; B cells and Plasma cells were defined by IGKC, JCHAIN, CD79A, CD27, CD74; NK and other immune cells were defined by CD4, CD8A, ITGAM, NKG7, KLRD1, PRF1, CD7, TRDC. The immune cell-enriched spots were defined by the average expression of the above immune markers higher than 0.9. The spots adjacent to immune cells were defined as the spots in contact with the immune cells. All the other spots were labeled as non-immune spots. In addition, HCC and benign liver spots were identified based on morphology in the H&E staining. Differential expression analyses were performed comparing (Jemal et al., 2012) HCC adjacent to versus away from immune cell-enriched spots (Jemal et al., 2012); Benign liver cells adjacent to versus away from immune cell-enriched spots (McGuire, 2016); HCC cells adjacent to immune spots versus benign liver cells adjacent to immune spots. Further, top genes were screened by integrating these differentially expressed genes with the top 3,000 high-variable genes. These genes were then used for Ingenuity Pathway Analysis (https://digitalinsights.qiagen.com/products-overview/discovery-insights-portfolio/analysis-and-visualization/qiagen-ipa/). The software performs enrichment tests between the selected gene markers and the pathway gene sets. It also calculates a z-score indicating the directionality of the pathways, with positive z-score for activated pathways, and negative z-scores for inhibited pathways. Per spatial spot, gene set variation analysis (GSVA) (Hanzelmann et al., 2013)was performed to calculate the enrichment score of the selected pathways, where a positive score indicates the activation of the pathway and a negative score implies the inhibition.

The spatial transcriptomics data were further integrated with the public single-cell RNA-seq data (Ma et al., 2022), which contains samples from the HCC tumor cores, tumor borders, and adjacent non-tumor tissues. Using this well-annotated scRNA-seq dataset as the reference, spatial deconvolution was performed and visualized by tool CARD (Ma and Zhou, 2022) to spatially infer the proportions of different immune cell types per immune spot. Deconvolution analysis was further applied on the immune spots to reveal the composition of six macrophage subtypes (Li et al., 2024). To investigate whether sequencing depth influences the overall myeloid composition, we applied the permutational multivariate ANOVA test on the center-log-ratio transformed myeloid composition data using R package vegan. In addition, cellular interaction analysis was performed using CellChat (Jin et al., 2021) to reveal the ligand-receptor interactions among various categories of immune cells located in the tumor and benign tissue regions. Common and unique interaction signaling pathways were compared between the deep and standard sequencing data.

### Data availability

The spatial transcriptomics data were submitted to the Gene Expression Omnibus (GEO) database with accession ID GSE283406. Scripts to analyze the spatial transcriptomics data were uploaded to GitHub: https://github.com/SilviaLiu12345/Spatial_transcriptomics_deep_vs_standard.

## Results

**FIGURE 1:**
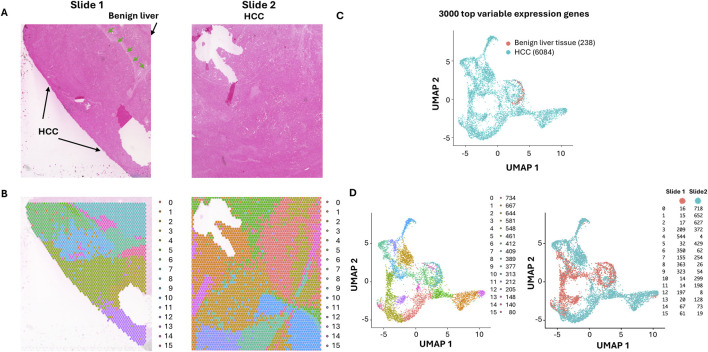
Spatial distribution of HCC clones with ultra-depth sequencing. **(A)** Hematoxylin and Eosin staining of HCC and its adjacent benign tissues. The areas of benign liver tissues and HCC are indicated. The demarcation between the benign liver and HCC is indicated by arrows. **(B)** The UMAP distribution of benign liver and HCC cells. The HCC and benign liver cell clusters are indicated. **(C)** Spatial visualization of cell clusters in slides. Each cluster distribution is indicated by its unique color. **(D)** UMAP spot distributions of 16 spatial clusters (left) and UMAP spot distributions of slides 1 and 2 (right).

To analyze the spatial transcriptomes and gene expression alteration patterns in a space-related fashion, we selected a case of moderately differentiated HCC containing multiple cancer nodules. As shown in Figure 1A, two slides were analyzed for the spatial gene expression. One of the slides contained some benign liver tissues adjacent to the cancer, while the other slide contained only liver cancer tissues. Deep sequencing was performed to reach 697,228 to 1,327,551 mean reads per spot, detecting 1,465 to 3,223 median genes per spot. Spots from the two slides were integrated and normalized together. Then clustering on these spots using Seurat single-cel clustering were performed to detect the tumor heterogeneity. Two slides account for 6,320 spots of tissues. A Seurat package was employed to identify marker genes that clustered the spots. Sixteen distinct clusters of spots were identified based on the top 3,000 highly variable gene expressions (Figures 1B–D; Supplementary Table S1). The distributions of these spot clusters on the slides did not appear random but rather aggregated in a distinct, patchy manner. Since most of the spots were cancer cells dominated. These clusters may reflect gene expression variations among the cancer cells. Pathway analyses (Supplementary Tables S2–S17) showed that the areas of benign liver tissues were dominated by gene expression of biosynthesis of cholesterol (Supplementary Table S11). They were mostly aggregated as a cluster (cluster 9, Figures 1B,D). On the other hand, high heterogeneity for the areas of HCC was identified: Fifteen distinctive clusters were found (Figures 1C,D). Some have characteristics of fibrotic gene expression patterns (clusters 0, 6, 14, and 15, Supplementary Tables S2, S8, S16, S17), while the others were also dominated by matrix/cell-cell contact activation pathways (clusters 3, 5, 8, 14, and 15, Supplementary Tables S5, S7, S10, S16, S17). Pro-growth pathways were found to overexpress in clusters 1, 3, 8, and 11 (Supplementary Tables S3, S5, S10, S13). Genes responsible for coagulation activation, one of the key features of HCC, were found overexpressed in clusters 2, 10, and 11 (Supplementary Tables S4, S12, S13). Many of the clusters overexpressed genes essential for oxidative metabolism (clusters 4, 5, 6, 7, 10, 12, and 13, Supplementary Tables S6–S9, S12, S14, S15). Their spatial distribution in the slides was aggregated in a contiguous patchy fashion, suggesting that they may arise as clonal expansions from single cells.

### Identification of immune cell-enriched spots

**FIGURE 2:**
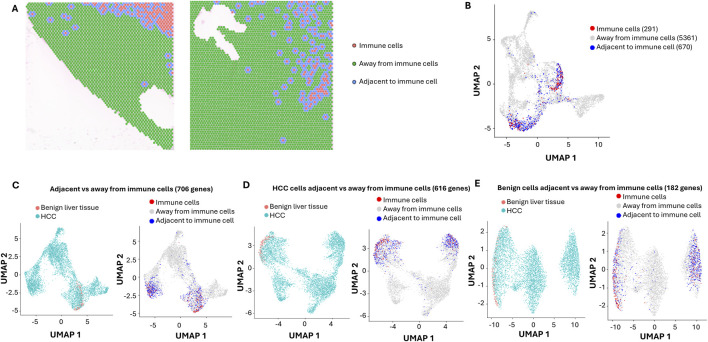
Impact of Immune cells on HCC and benign liver cells with ultra-depth sequencing. **(A)** Spatial visualization of immune cell-enriched, non-immune HCC, and benign liver cell spots. Immune cell-enriched spots are indicated in pink, while cells immediately adjacent to immune spots are labeled in blue. All cells away from immune spots are labeled in green. **(B)** The distributions of immune cell-enriched spots and cells adjacent to immune spots in UMAP clusters. **(C)** UMAP distributions of HCC and benign liver cells based on 706 differential expressed genes between cells adjacent to and away from immune spots (left) or UMAP distributions of immune spots, spots away from immune cells, and spots adjacent to immune cells (right). **(D)** UMAP distributions of HCC and benign liver cells based on 616 differential expressed genes between HCC cells adjacent to and away from immune spots (left) or UMAP distributions of immune spots, spots away from immune cells, and spots adjacent to immune cells (right). **(E)** UMAP distributions of HCC and benign liver cells based on 182 differential expressed genes between benign cells adjacent to and away from immune spots (left) or UMAP distributions of immune spots, spots away from immune cells, and spots adjacent to immune cells (right).

The cancer microenvironment plays a critical role in shaping cancer development. Significant lymphocytes and macrophages were identified in both benign liver cancer and HCC areas. To investigate the impact of these immune cells on liver cancer, selected gene markers for cells of immune lineages including Kuffler cells (Cd68, Cd163, Lyz, C1qa, Aif1), T cells (Cd3d, Cd2, Il7r, Trbc2, Cd69, Cd4, Cd8, Cd11), B cells (Igkc, Jchain, Cd79a, Cd27, Cd74) and NK (Nkg7, Klrd1, Prf1, Cd7, Trdc) cells were analyzed for each spot. Spots with average expression of these markers above 0.9 were deemed immune cell-enriched spots. Two hundred eighty-nine spots were identified as immune cell-enriched. As shown in Figure 2A, many immune cell-enriched spots were located in the benign liver tissues adjacent to HCC, while the distribution of immune cell-enriched spots in the HCC area varied from region to region. Interestingly, all types of immune cells were more abundant in the immune cell-enriched spots in the benign liver (Supplementary Figure S2). Most immune cell-enriched spots co-localized with clusters 3 and 9 (Figure 2B). Next, we categorized the immune microenvironment into cells that were adjacent to immune cell-enriched spots (adjacent to immune cells) versus those that were away from the immune cells (away from immune cells) (Figures 2A,B). Six hundred seventy spots were deemed impacted by location adjacent to immune cells, including 84 spots from benign liver and 586 from HCC. Spots away from immune cell-enriched spots were 5,361.

### Impact of immune microenvironment on HCC cells

To investigate the impact of immune cells on their surrounding microenvironment, differential expression analyses were performed to identify the variable genes between the spots adjacent to immune cells and the spots away from immune cells. Seven hundred and six genes were found to have differential expressions between the two groups (Figure 2C). When analyzing HCC cells’ responses to immune cell-enriched spots, 616 genes were found to be differentially expressed (Figure 2D). While the HCC cells adjacent to immune cell-enriched spots were down on lipid metabolism, post-translational protein phosphorylation pathways, acute phase response, integrin cell surface interaction, and extracellular matrix organization pathways were up (Supplementary Table S18). For benign liver cells adjacent to immune cell-enriched spots, only 182 genes were differentially expressed versus benign liver cells away from the immune cell-enriched spots (Figure 2E). Benign liver cells adjacent to immune cell-enriched spots have downregulation of genes in acute phase response, lipid metabolism, and post-translational protein phosphorylation pathways, while upregulated in IL12 signaling and neutrophil extracellular trap signaling pathways (Supplementary Table S19), reflecting responses to cytokine release from the immune cells. Interestingly, the differences in response to immune cells between HCC and benign liver lie in the dramatic upregulation of integrin interaction and acute phase response in the HCC cells (Supplementary Table S20; Supplementary Figure S1). Hepatic fibrotic/stellate activation pathways showed a dominant presence in the benign liver but a somewhat heterogeneous distribution in the HCC regions. In addition, downregulation of gene expressions in the molecular mechanism of cancer and Rho GTPase pathways in the HCC cells were some of the prominent features of the pathway changes (Supplementary Table S20). These findings suggest that the primary role of immune cells is to shut down the cancer signaling pathways in liver cancer but to spare such impact in the benign liver.

### The lack of immune related signaling activity in HCC regions

**FIGURE 3:**
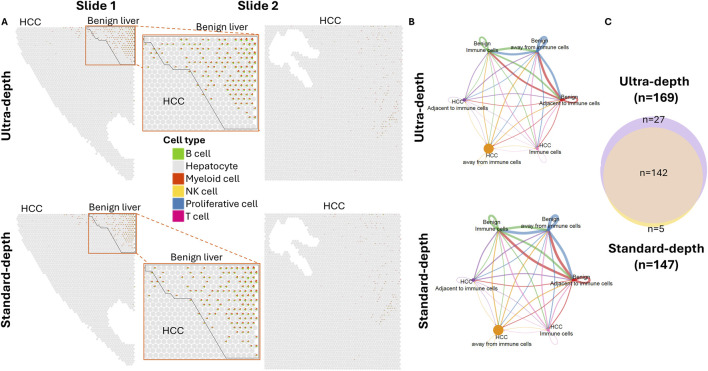
Spatial cell-cell interaction based on immune impact. **(A)** Spatial visualization of immune cell composition in slides. HCC and benign liver areas are indicated. Each cell type is indicated by the colorization of a miniature pie chart. Top panel: Ultra-depth sequencing; Bottom: Standard-depth sequencing. **(B)** Cell-cell communications of subpopulations of cells away or adjacent to immune-enriched spots. Top: ultra-depth sequencing; bottom: standard-depth sequencing. **(C)** Venn diagram active cell-cell interaction signaling pathways in ultra-depth or standard-depth sequencing.

B cells and myeloid cells were the predominant cell types in the immune cell-enriched spots in both HCC and benign liver areas (Figure 3A; Supplementary Figure S2). Cell-cell interaction analyses suggested that 169 ligand-receptor pathway activations were impacted by immune cells (Figures 3B,C; Supplementary Tables S21–S22). In general, there was significantly less communication between cancer cells in comparison with the benign liver cells. EGF pathway has been well established to play a crucial role in HCC development. Our analysis showed that HCC cells either away or adjacent to immune cells were activated by EGF produced from benign hepatocyte, albeit on a significantly smaller scale in comparison with the benign liver cells (Supplementary Figure S3A). On the other hand, HCC cells were the initiators for the IGF pathway. Interestingly, HCC cells either away from or adjacent to immune enrichment spots showed little activation of immune regulatory signaling such as CD23, CD39, CD86, CD96, IL2, IL17 and MHC-I, in contrast to robust communications of immune signaling between the benign liver cells (Supplementary Figures S3B, S3C). The lack of immune pathway activity in the cancer cells indicated an immunological evasion of HCC cells. In contrast to the immune response, fibrotic reactions were present universally in both benign and cancer regions of the samples (Supplementary Figure S4), suggesting a cirrhotic background and etiology.

### Analysis of standard-depth sequencing

**FIGURE 4:**
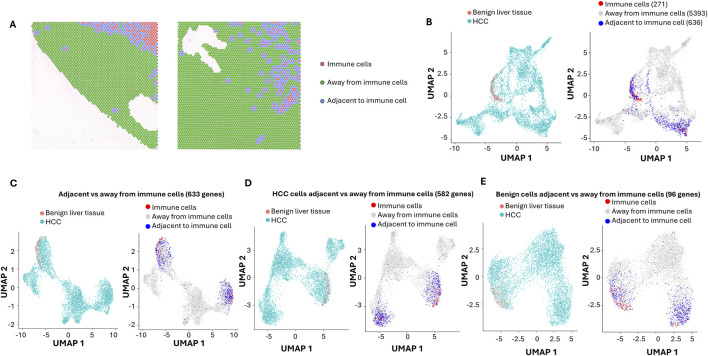
Impact of Immune cells on HCC and benign liver cells with standard-depth sequencing. **(A)** Spatial visualization of immune cell-enriched, non-immune HCC, and benign liver cell spots. Immune cell-enriched spots are indicated in pink, while cells immediately adjacent to immune spots are labeled in blue. All cells away from immune spots are labeled in green. **(B)** The distributions of immune cell-enriched spots and cells adjacent to immune spots in UMAP clusters. **(C)** UMAP distributions of HCC and benign liver cells based on 633 differential expressed genes between cells adjacent to and away from immune spots (left) or UMAP distributions of immune spots, spots away from immune cells, and spots adjacent to immune cells (right). **(D)** UMAP distributions of HCC and benign liver cells based on 582 differential expressed genes between HCC cells adjacent to and away from immune spots (left) or UMAP distributions of immune spots, spots away from immune cells, and spots adjacent to immune cells (right). **(E)** UMAP distributions of HCC and benign liver cells based on 96 differential expressed genes between benign cells adjacent to and away from immune spots (left) or UMAP distributions of immune spots, spots away from immune cells, and spots adjacent to immune cells (right).

Standard-depth sequencing was performed as a control to analyze the impact of ultra-depth sequencing. The mean reads in our standard-depth sequencing were 17,914–39,237 per spot. The median numbers of genes identified per spot were 1,322 to 2,769. Using the same algorithm as described for the ultra-deep sequencing (top 3,000 variable genes, Supplementary Table S23), 15 distinct clusters were identified (Supplementary Figure S5; Supplementary Tables S24–S38). Most of these clusters were similar in terms of pathway distributions to those identified by ultra-depth sequencing. Benign liver cells were only limited to cluster 9. When immune markers were analyzed (Figures 4A,B), the standard-depth produced fewer immune cell-enriched spots than the ultra-depth (271 vs. 289). Only 633 genes were found differentially expressed between cells adjacent to and away from the immune cell-enriched spots (versus 706 genes for ultra-depth, Figure 4C). When HCC adjacent to immune cell-enriched spots were analyzed in comparison to HCC cells away from the immune cell-enriched spots, standard-depth sequencing showed fewer genes (582 vs. 616, Figure 4D) and pathway affected (Supplementary Table S39, 420 versus 462). In addition, standard-depth sequencing also showed fewer genes (96 versus 182, Figure 4E) and pathways (Supplementary Table S40, 84 versus 137) impacted by the immune cells in the benign liver tissues. Some of the pathways showed opposite directions between standard-depth and ultra-depth sequencing. These results suggest that ultra-depth sequencing may significantly improve the analysis.

**FIGURE 5:**
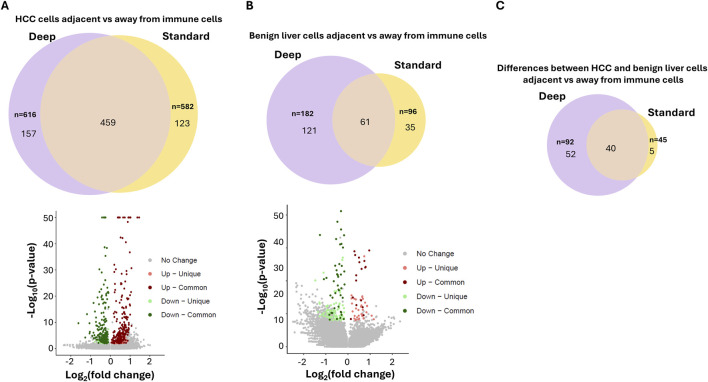
Impact of ultra-depth sequencing on differentially expressed gene discovery. **(A)** Venn diagram (top) and Volcano plot (bottom) of the differentially expressed genes between HCC cells adjacent to versus away from immune cells with ultra-depth or standard-depth sequencing. **(B)** Venn diagram (top) and Volcano plot (bottom) of the differentially expressed genes between benign liver cells adjacent to versus away from immune cells with ultra-depth or standard-depth sequencing **(C)** Venn diagram of the differences of differentially expressed genes between HCC and benign liver cells adjacent to vs. away from immune cells.

To investigate what differential genes were identified between these two approaches, the genes from standard-depth sequencing were matched with those from ultra-depth sequencing. Through Kolmogorov-Smirnov test, 14,069 of 18,085 (78%) genes were found to have an increased % spot detection by ultra-depth sequencing (Supplementary Table S41). One of the top genes detected more often in ultra-depth sequencing is ISG15, a ubiquitin-like protein involved in chemotactic and cancer-promoting signaling (Meng et al., 2024; Kang et al., 2022). Ultra-depth sequencing detected numerous spots with increased read counts for ISG15 expression missed by standard sequencing (Supplementary Figure S6). When immune-impacted spots were analyzed, 25% (157/616) genes of ultra-depth sequencing from HCC regions impacted by immune cell-enriched spots were not identified by standard-depth (Figure 5; Supplementary Tables S42–S45). Surprisingly, 123 genes identified through standard-depth were not found in ultra-depth. For benign liver regions, 66% (121/182) of genes of ultra-depth were not found in the standard-depth sequencing. When the differences between HCC and benign liver immune-impacted genes were analyzed, only 43% of ultra-depth genes were found in the standard-depth. These results indicate that ultra-depth sequencing uncovered large numbers of biologically significant genes and pathways that standard-depth sequencing did not find. Indeed, some of the immune evasion signaling in HCC cells, such as PD-L1, CD45, CD80, etc. were only uncovered by ultra-depth sequencing (Supplementary Figure S3C). On the other hand, some of the most impacted genes by immune cells detected in both ultra-depth and standard sequencings are carrier proteins such as retinol binding protein 4 and albumin (Supplementary Figures S7–S9). Both carrier proteins showed significant downregulation in cells (both HCC and benign liver) adjacent to immune cells.

## Discussion

HCC is highly heterogeneous. The genotype of HCC may vary from region to region. Our study confirmed that large variations in gene expressions occurred in different regions of the cancer. Such variations were quite distinct from each other, suggesting that subclones of cancer cells had significant evolution from their origin. Underlying these gene expression variations were probably new genome mutations or chromosomal rearrangements. Indeed, recent long-read single-cell sequencing suggests that extensive mutation evolution occurred in a small region of HCC (Liu et al., 2024a). The mutation evolution may drive the gene expression alterations that produce the cancer phenotype (Liu et al., 2021). The exact mechanisms that induce the genetic mutations remain elucidated. However, it is likely that the DNA repair mechanism in HCC is defective. Such a defect may lead to a cascade of mutation accumulation and changes in gene expression patterns.

The tumor microenvironment has long been known to impact cancer development. Our study showed significant immune cells infiltrating both the cancer and benign liver areas. An interesting finding of myeloid cell-enriched spots is the dominance of type 5 macrophage spots based on macrophage subtype marker analysis (Li et al., 2024) (Supplementary Figure S10). The significance of the homogeneity of macrophages is not immediately clear, but the lack of diversity may imply a pathological process in cancer development. To further examine how sequencing depth associates with the myeloid composition deconvolution, we applied the permutational multivariate ANOVA test on the center-log-ratio transformed composition of Macrophage type 1 to type 6. The results indicated that both sequencing depth (deep vs. standard) and immune spots region (HCC vs. benign) have significant effects on the myeloid profiles (p = 0.016 and p = 0.001, respectively). However, the interaction of these two factors has no significant correlation (p = 0.932). In the regions near the immune cell-enriched spots, many genes showed distinct responses to the presence of these immune cells. There were significant differences in response to immune cells between benign liver and HCC. There were 3-fold more genes and pathways altered in HCC in comparison with benign liver cells in response to immune cells, even though the immune cells were less abundant in the immune cell-enriched spots of the HCC area. One of the qualitative differences between the HCC and benign liver responses to immune cells is the genes of the acute response phase pathway: Genes such as SOD2 or ORM1 were upregulated in HCC but downregulated in benign liver. These differential responses to immune cells may suggest an innate difference in the mechanism of cytokine signaling between cancer and benign liver cells. These differences may result from the differences in the immune/somatic cell interaction or the differences in cellular sensitivity to cytokines secreted by the lymphocytes. The lack of vigorous responses from the benign liver tissues can be interpreted as normal immune adaptation.

Ultra-depth sequencing appears to offer significant advantages in identifying differentially expressed genes and pathways, particularly if the gene expression levels are not very high. One interesting finding is that ultra-depth sequencing did not cover all the differentially expressed genes discovered by 10-fold lower-depth sequencing. This suggests that even ultra-depth sequencing does not escape significant sampling errors. However, the sampling error rate could be higher in standard-depth sequencing. Neither ultra-depth nor standard-depth sequencing eliminates false negative discoveries. One potential risk for ultra-depth sequencing is that it may over disperse and induce overinterpretation of the data. Thus, new analytical tools may be needed to address these potential risks. Even though the current study was limited to one case of HCC study, ultra-depth sequencing did produce significantly more differentially expressed genes and thus uncovered more mechanisms that are important to understand the spatial relationship and interaction between immune and cancer cells, or between cancerous and benign cells. As we reach the era of ultra-affordable sequencing, ultra-depth spatial sequencing may present an important opportunity to decipher the mechanisms of cancer development.

## Data Availability

The datasets presented in this study can be found in online repositories. The names of the repository/repositories and accession number(s) can be found in the article/Supplementary Material.

## Supplementary material

SUPPLEMENTARY FIGURE S1 Heatmaps of top differential pathways between HCC and benign liver cells adjacent to immune cell enriched spots. **(A)** Ultra-depth sequencing. **(B)** Standard-depth sequencing.

SUPPLEMENTARY FIGURE S2 Immune cell composition is immune cell-enriched spots of HCC and benign liver. Average fractions of each type of immune cells of the immune cell-enriched spots were shown. Left: Ultra-depth sequencing; Right: Standard-depth sequencing.

SUPPLEMENTARY FIGURE S3 Cell-cell interaction and communication among subpopulations of benign liver and HCC cells impacted by immune cells. **(A)** EGF and IGF pathway communications among different groups of liver cells discovered by both ultra-depth and standard-depth sequencings. **(B)** Immune-related pathway communications among different groups of liver cells discovered by both ultra-depth and standard-depth sequencings. **(C)** Immune-related pathway communications among different groups of liver cells discovered by ultra-depth sequencing only.

SUPPLEMENTARY FIGURE S4 Spatial cell-cell interaction based on immune and fibroblast impact. (A) Spatial visualization of immune cell composition in slides. HCC and benign liver areas are indicated. Each cell type is indicated by the colorization of a miniature pie chart. Top panel: Ultra-depth sequencing; Bottom: Standard-depth sequencing.

SUPPLEMENTARY FIGURE S5 Spatial distribution of HCC and benign liver clusters with standard-depth sequencing. **(A)** Spatial visualization of cell clusters in slides. Each cluster distribution is indicated by its unique color. **(B)** UMAP spot distributions of 15 spatial clusters (top) and UMAP spot distributions of slide 1 and slide 2 (bottom).

SUPPLEMENTARY FIGURE S6 Spatial distribution of differences in read detection by ultra-depth sequencing over standard-depth sequencing. The heat maps were based on Log_2_ (read counts from ultra-depth+1)/(read counts from standard-depth+1). The numerical value is reflected by the variation of the color bar. Top representative genes were chosen based on their adjusted p-value from Supplementary Table S41.

SUPPLEMENTARY FIGURE S7 Spatial expression of top differential expression genes impacted by immune cell enriched spots. **(A)** Heat map of top 5 differentially expressed genes in HCC impacted by immune cells through ultra-depth sequencing. **(B)** Heat map of top 5 differentially expressed genes in HCC impacted by immune cells through standard-depth sequencing. **(C)** Heat map of top 5 differentially expressed genes in benign liver cells impacted by immune cells through ultra-depth sequencing. **(D)** Heat map of top 5 differentially expressed genes in benign liver cells impacted by immune cells through standard-depth sequencing.

SUPPLEMENTARY FIGURE S8 UMAP distribution of top differential expression genes impacted by immune cell enriched spots. **(A)** Expression distribution of top 5 differential expression genes in HCC impacted by immune cell enriched spots in UMAP through ultra-depth sequencing. **(B)** Expression distribution of top 5 differential expression genes in HCC impacted by immune cell enriched spots in UMAP through standard-depth sequencing. **(C)** Expression distribution of top 5 differential expression genes in benign liver impacted by immune cell enriched spots in UMAP through ultra-depth sequencing. **(D)** Expression distribution of top 5 differential expression genes in benign liver impacted by immune cell enriched spots in UMAP through standard-depth sequencing.

SUPPLEMENTARY FIGURE S9 Distribution of differential expressed genes in HCC and benign liver samples. UpSet plot was utilized to display the differential expressed pathways in HCC and benign liver cells impacted by immune cells.

SUPPLEMENTARY FIGURE S10 Distribution of subtypes of macrophages in myeloid cell-enriched spots. All myeloid cell-enriched spots were deconvoluted based on gene expression markers of M1 through M6 macrophages. The proportions of these macrophages were plotted for each myeloid cell-enriched spot. The color of each type of macrophage was indicated.
