# Supplementary material for: Deep spatial sequencing revealing differential immune responses in human hepatocellular carcinoma
Source: Front Cell Dev Biol. 2025 Jun 3;13:1600129. doi: 10.3389/fcell.2025.1600129 (PMC12170511; doi:10.3389/fcell.2025.1600129)

Supplemental figure 1A

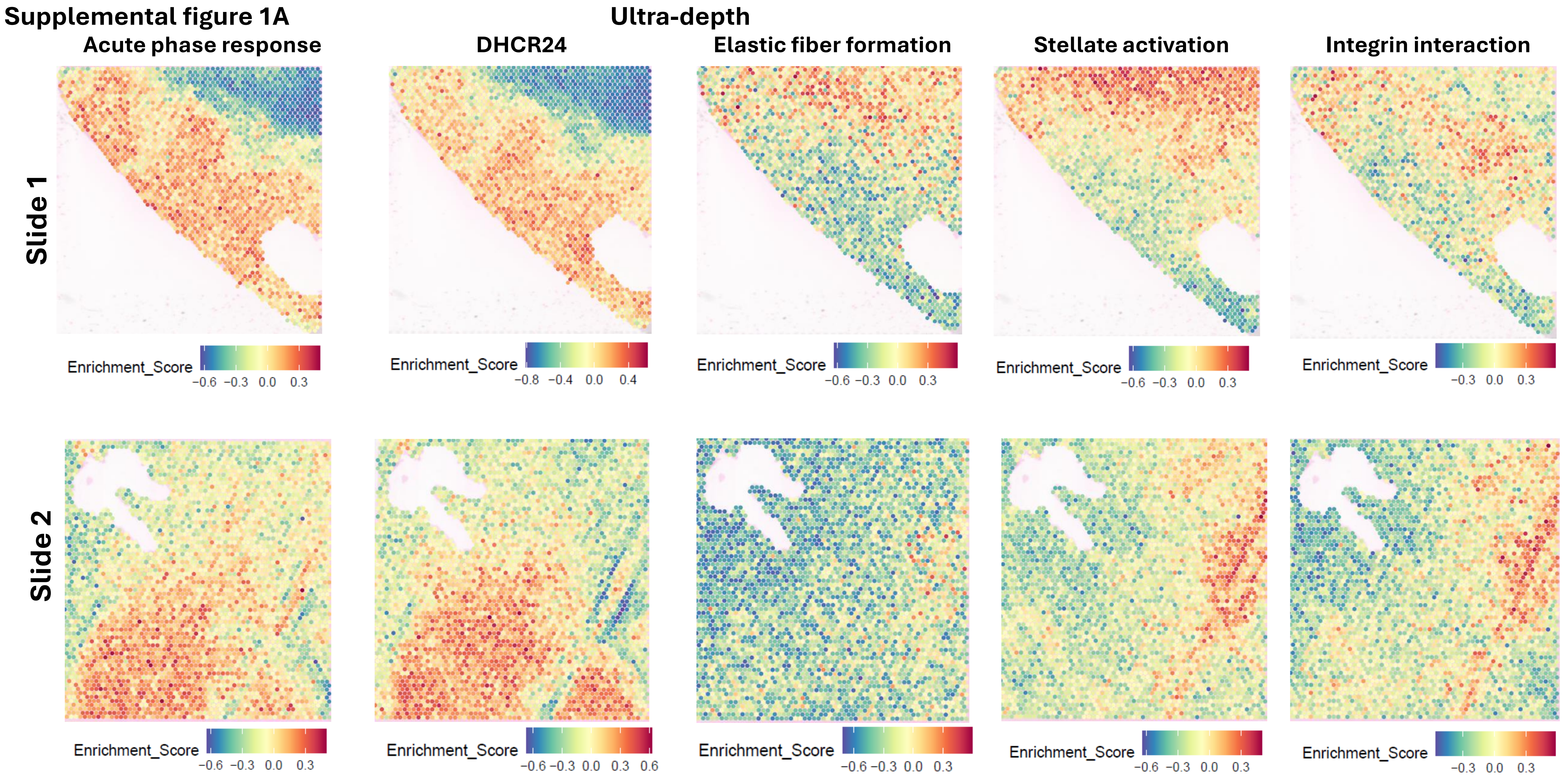

Supplemental figure 1A

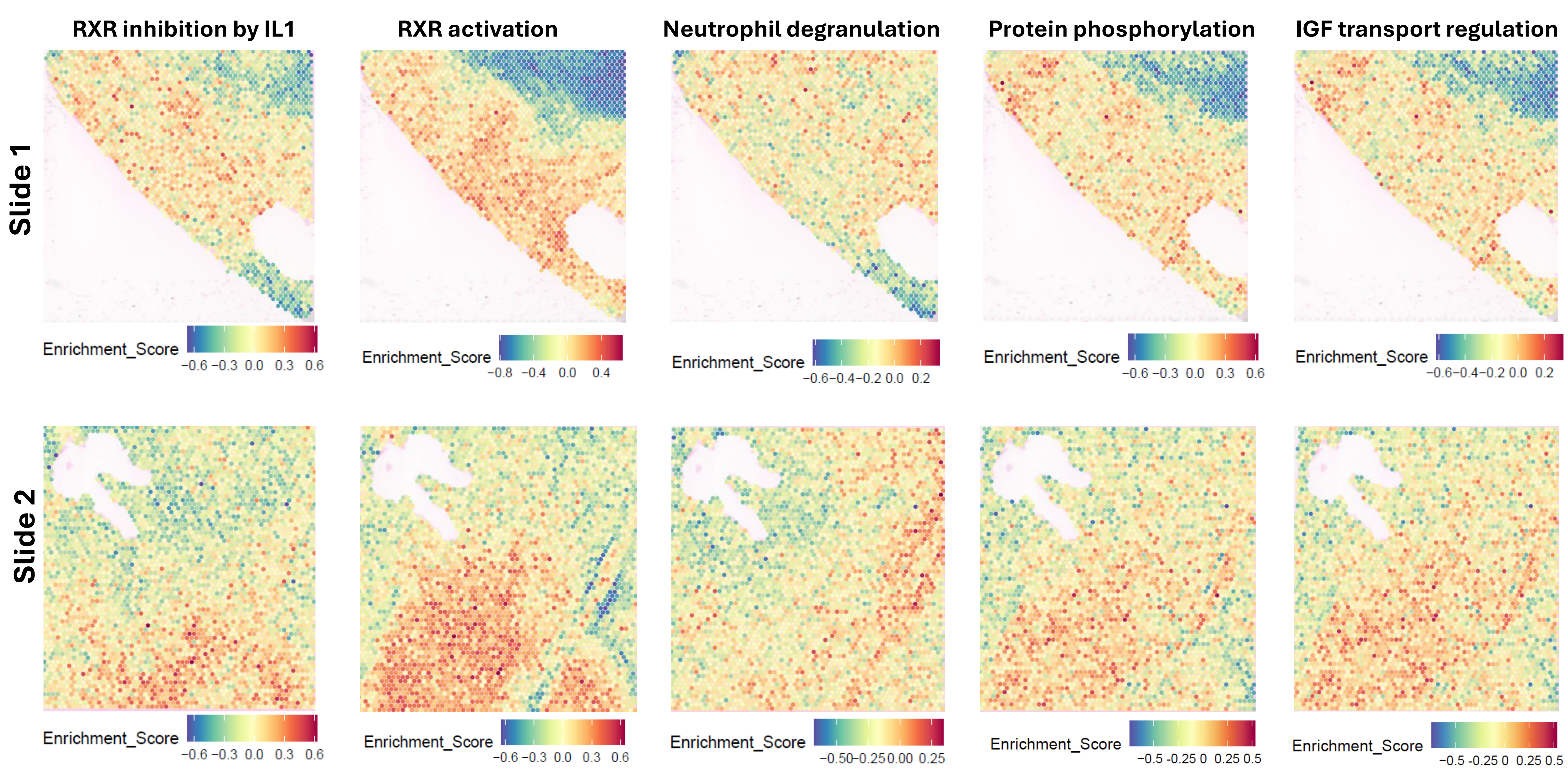

Supplemental figure 1B

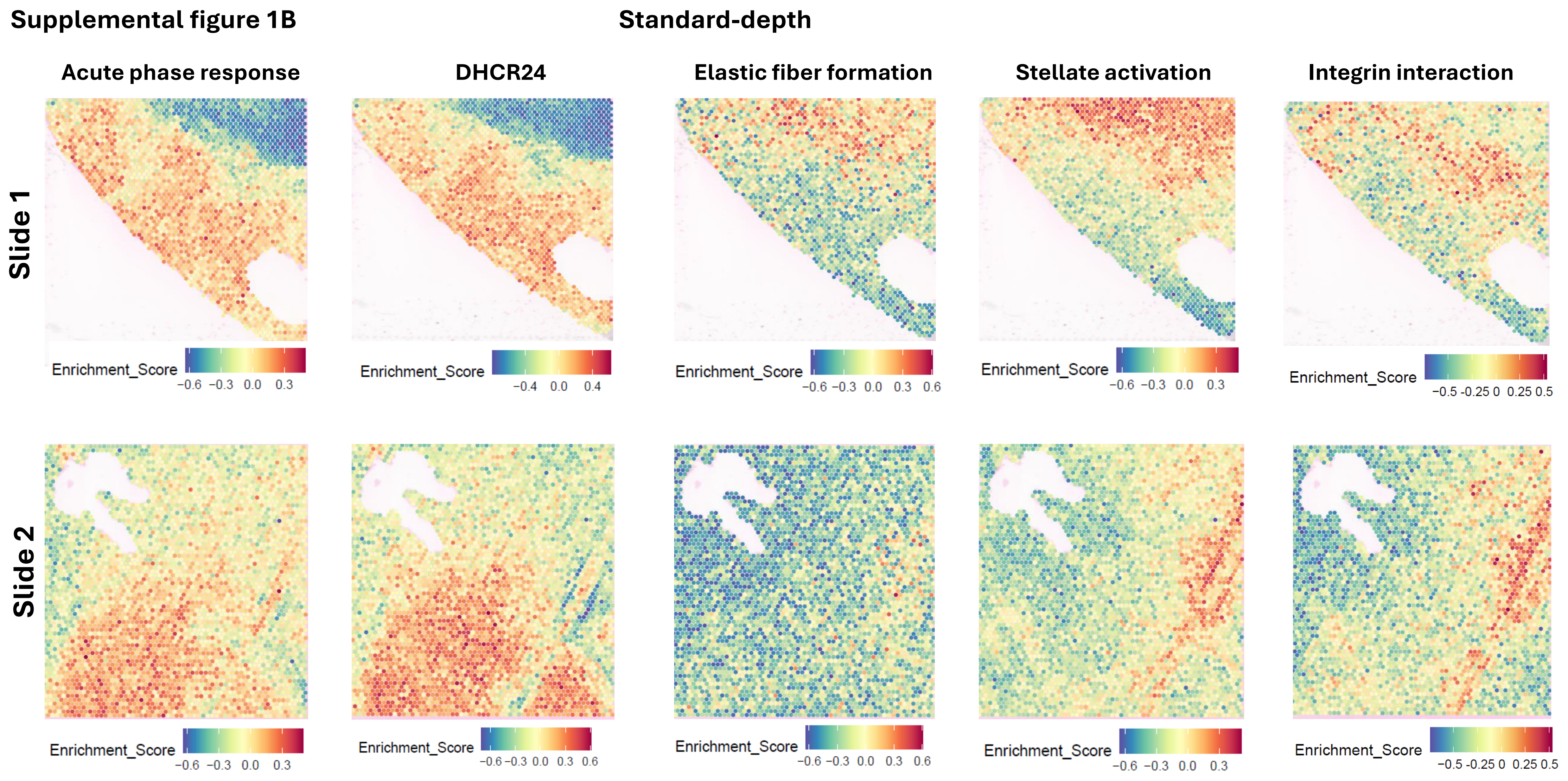

Supplemental figure 1B

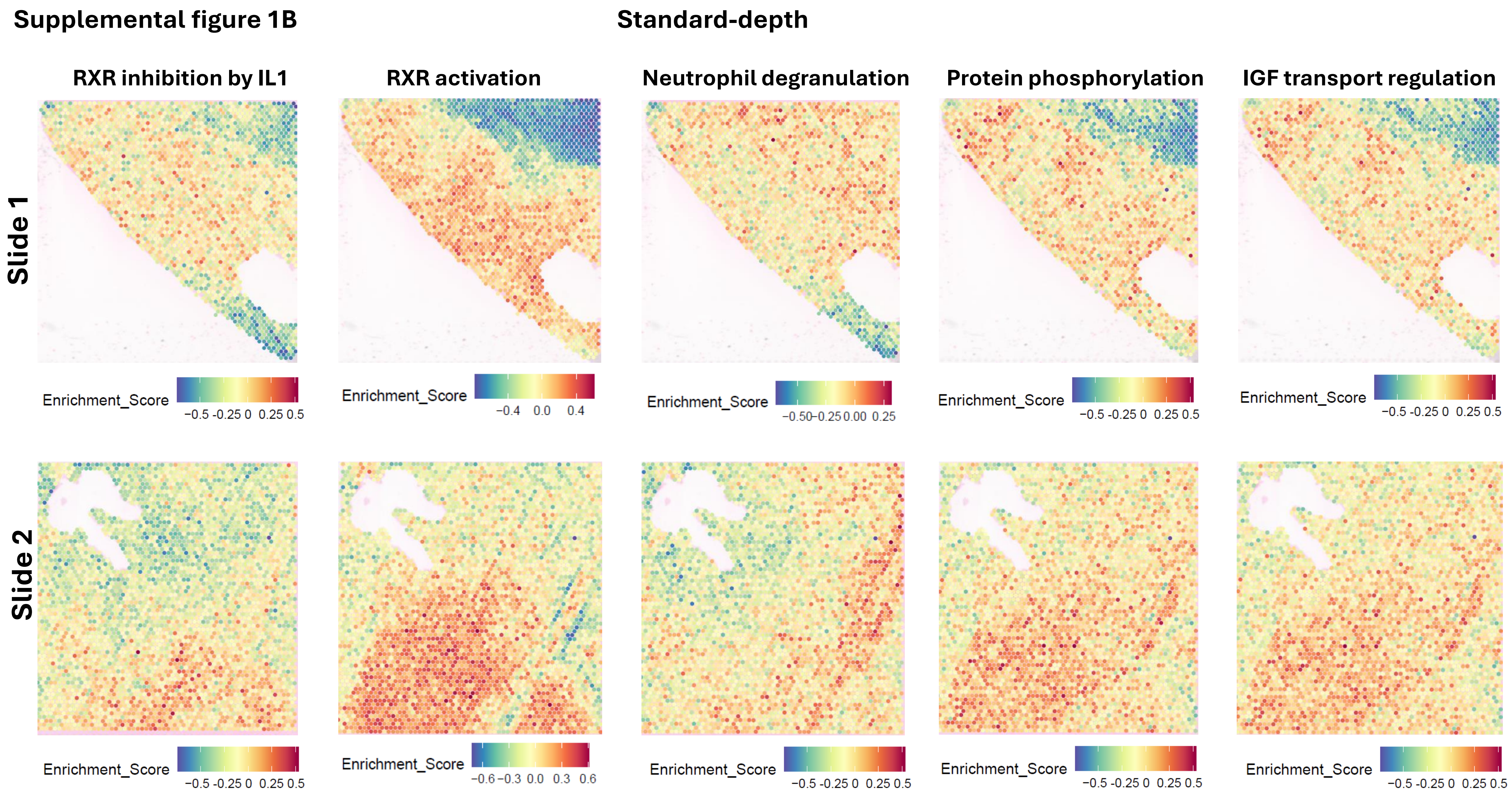

Supplemental figure 2

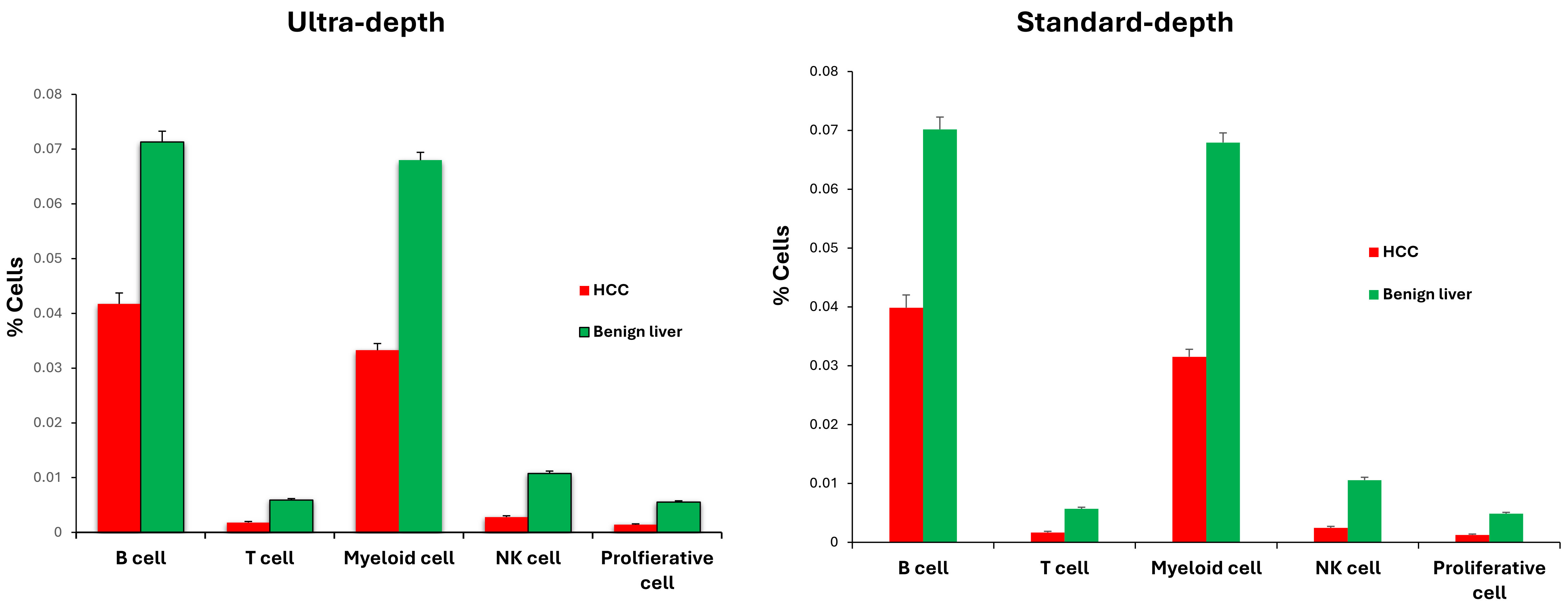

Supplemental figure 3A

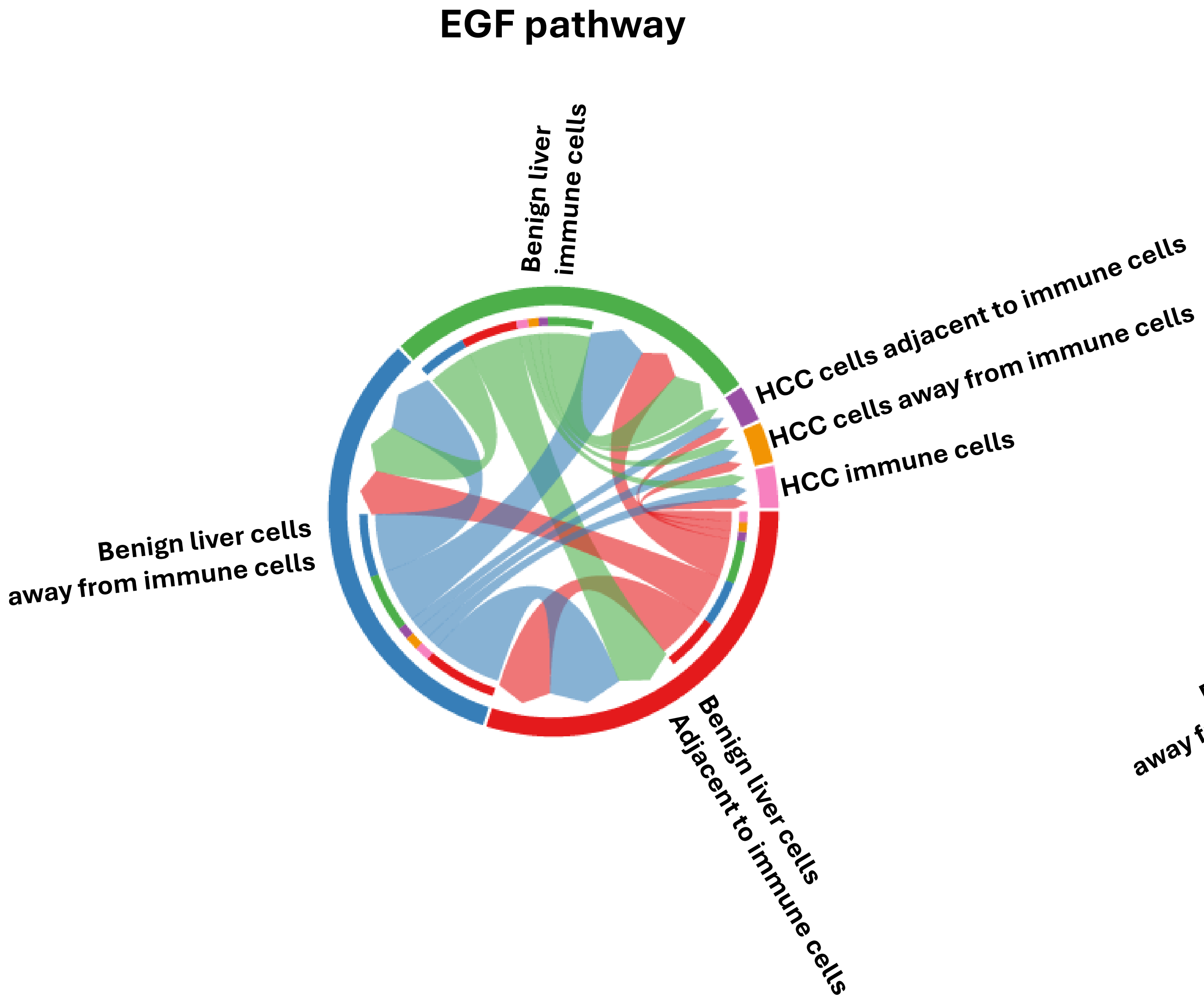

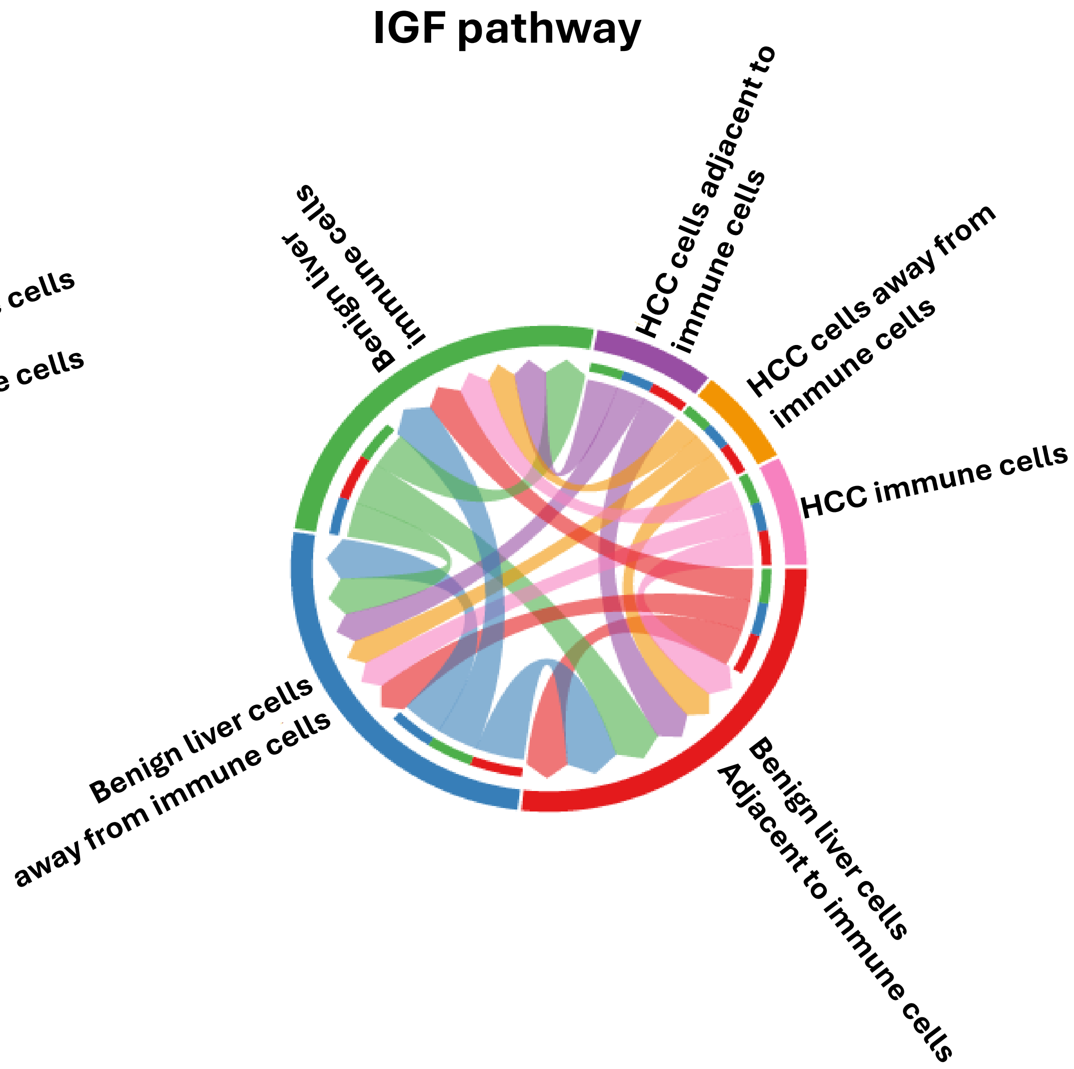

Supplemental figure 3B

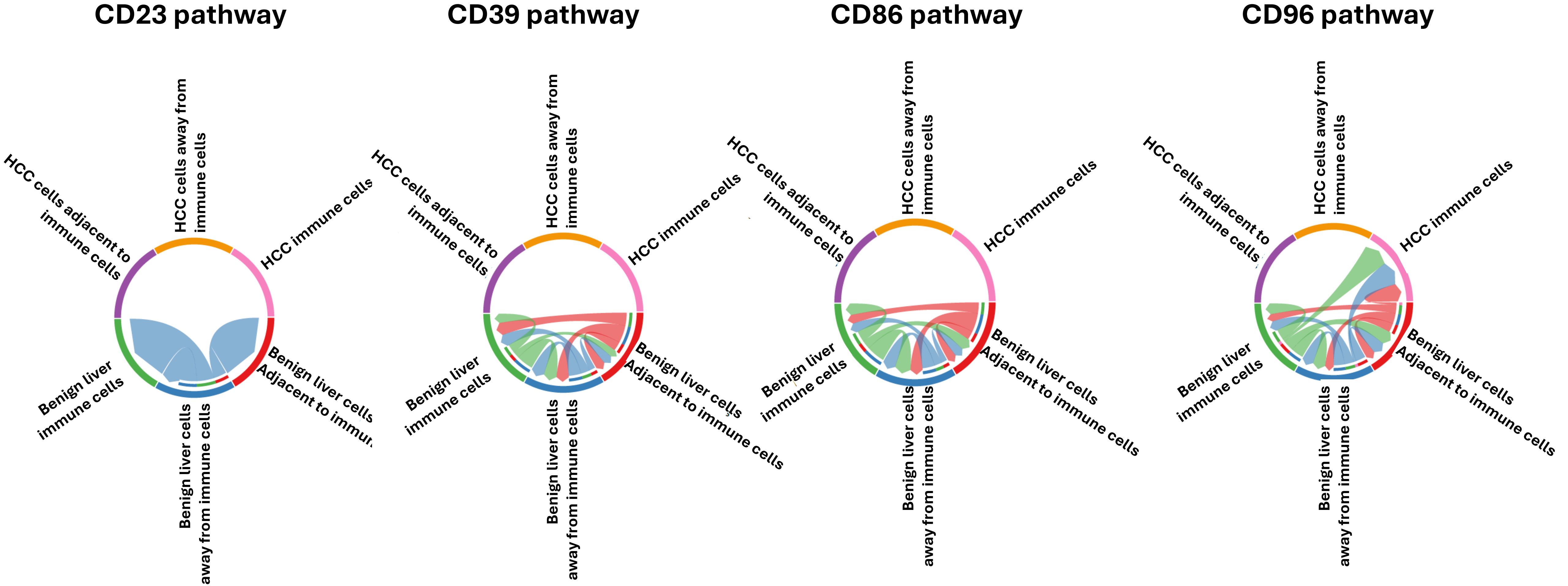

Supplemental figure 3B

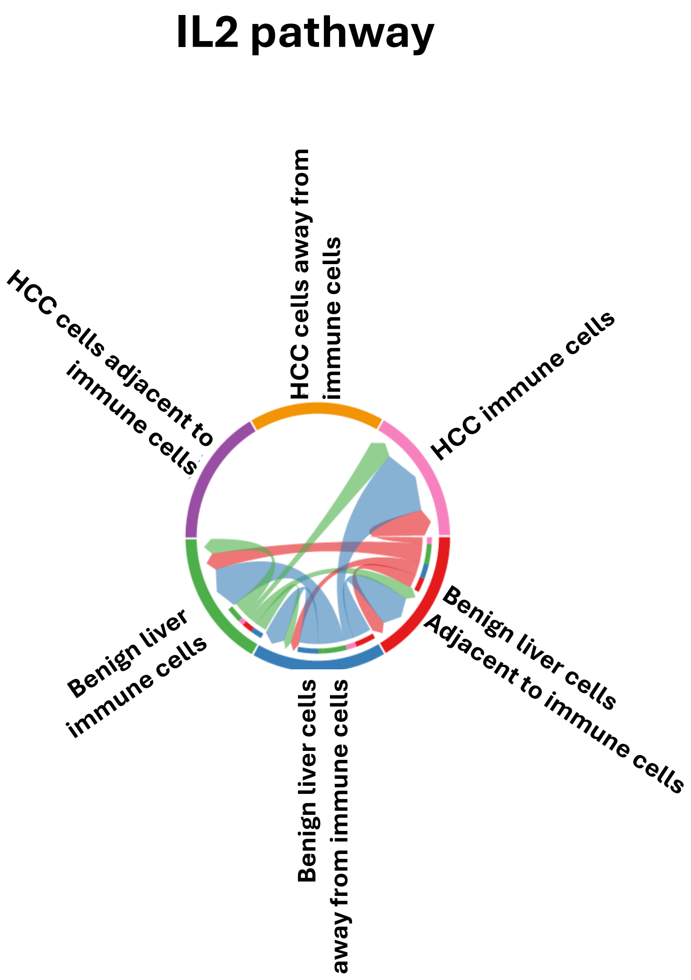

Supplemental figure 3C

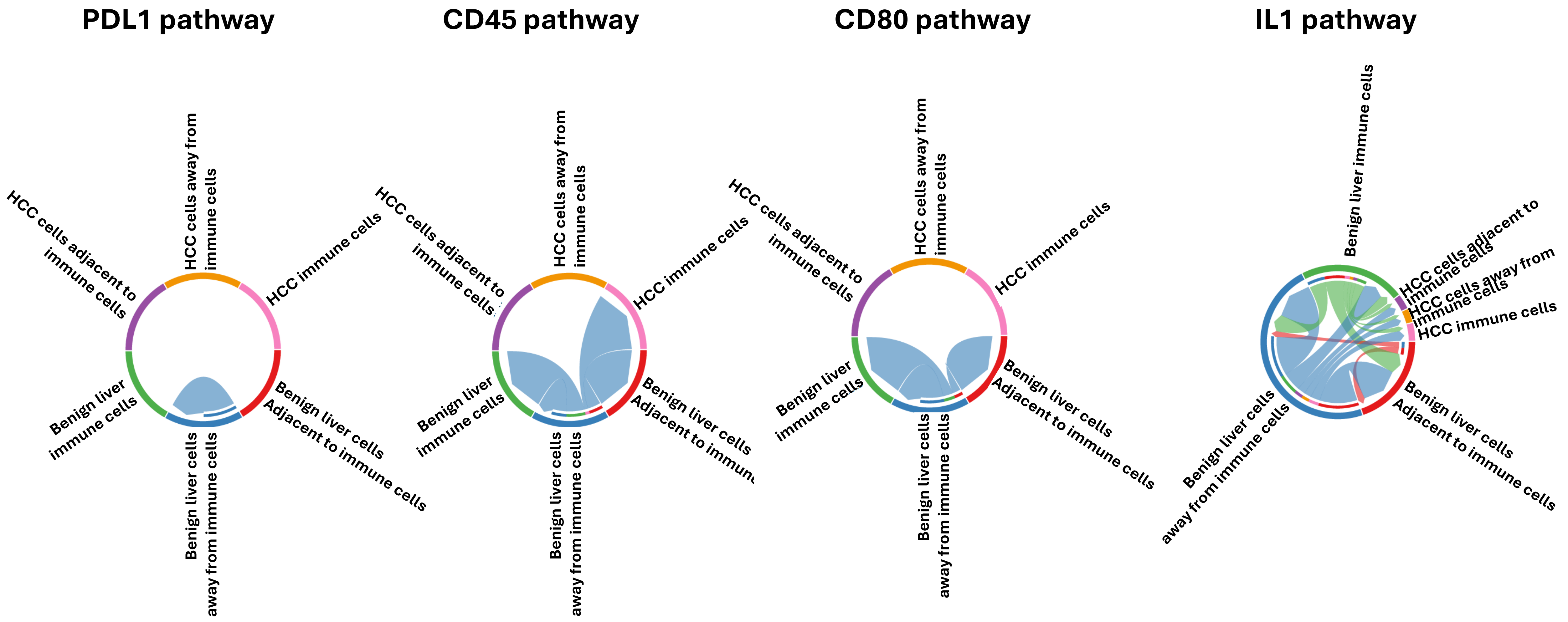

Supplemental figure 4

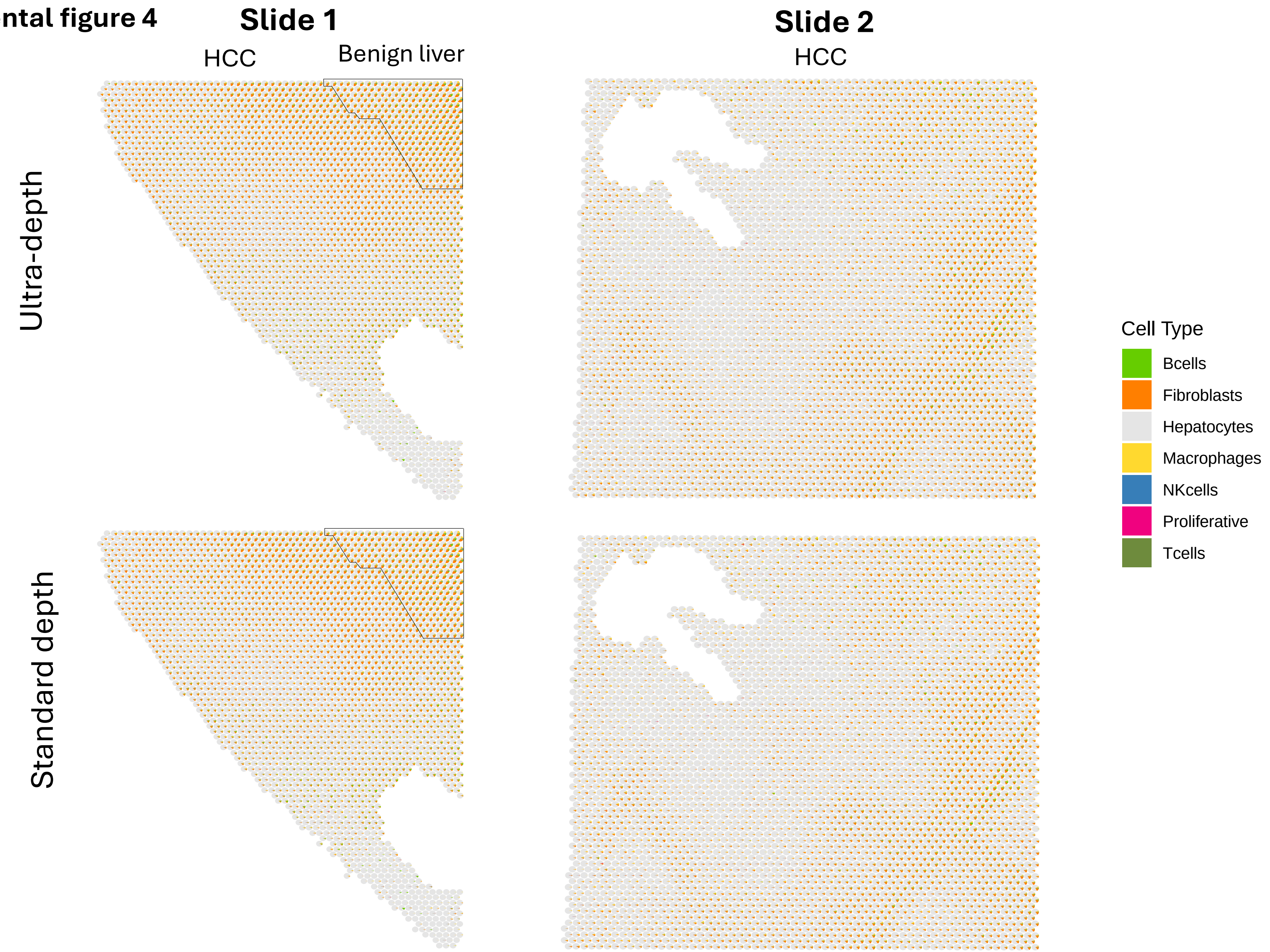

Supplemental figure 5

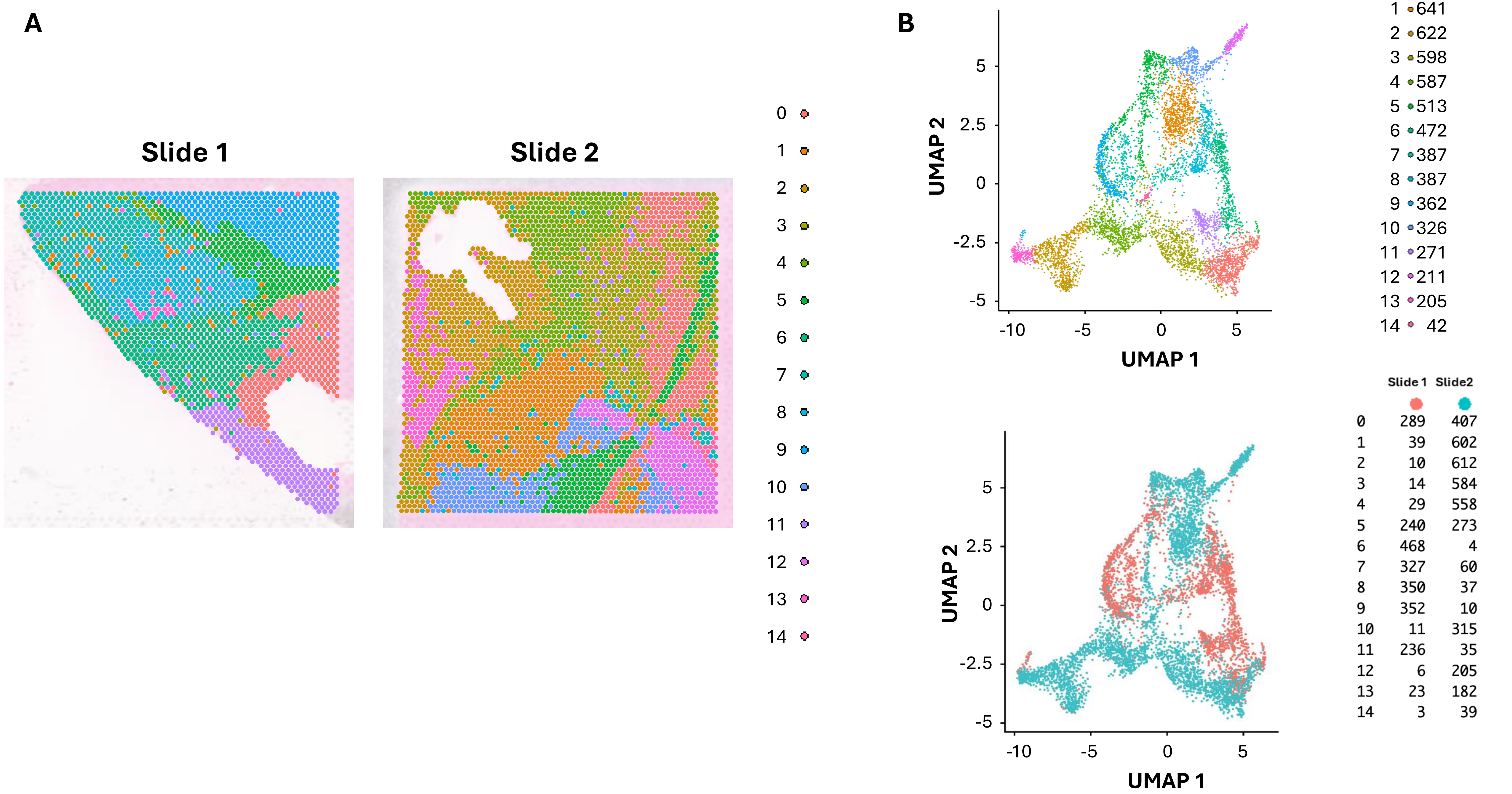

Supplemental figure 6

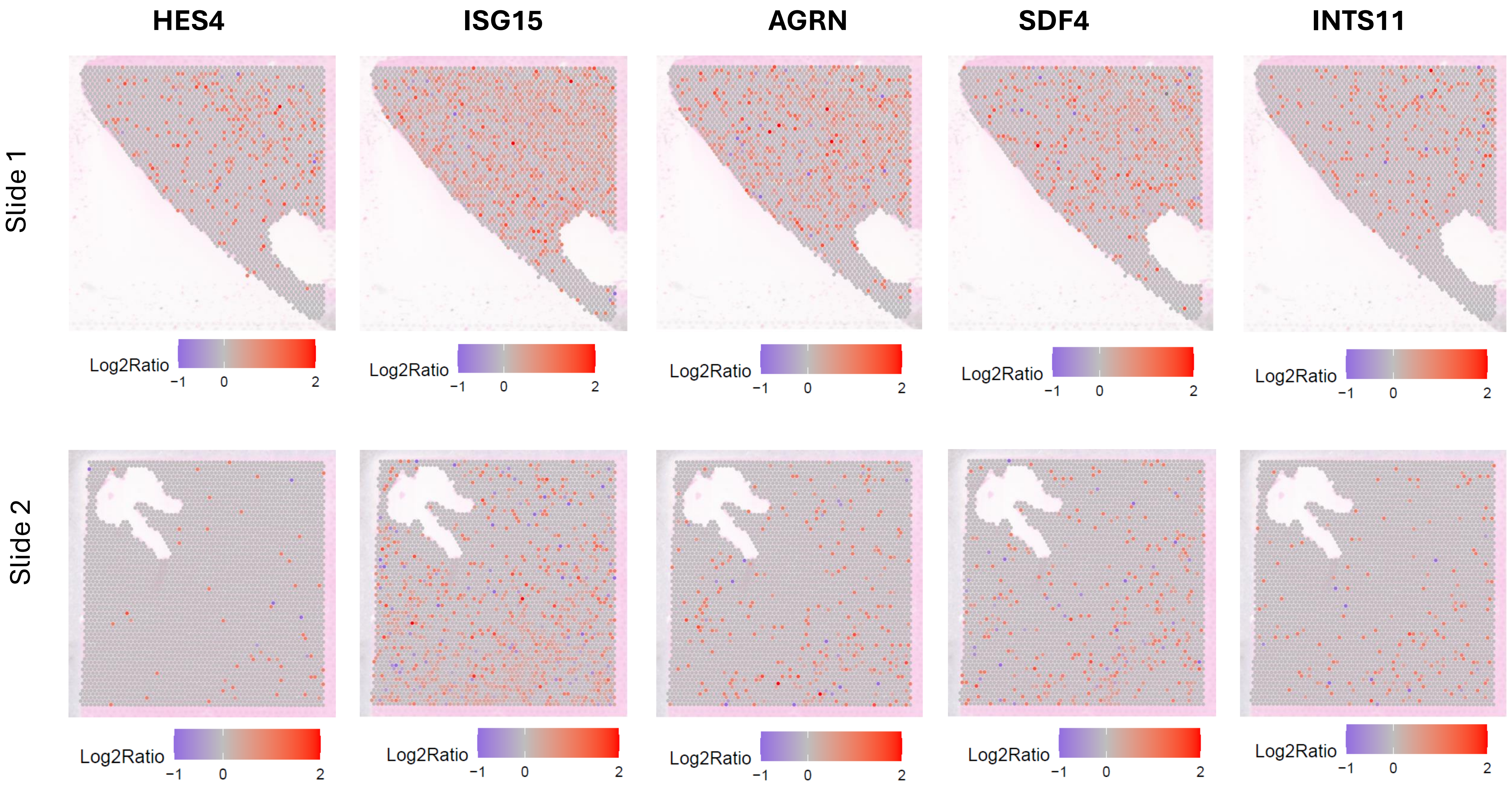

Supplemental figure 6

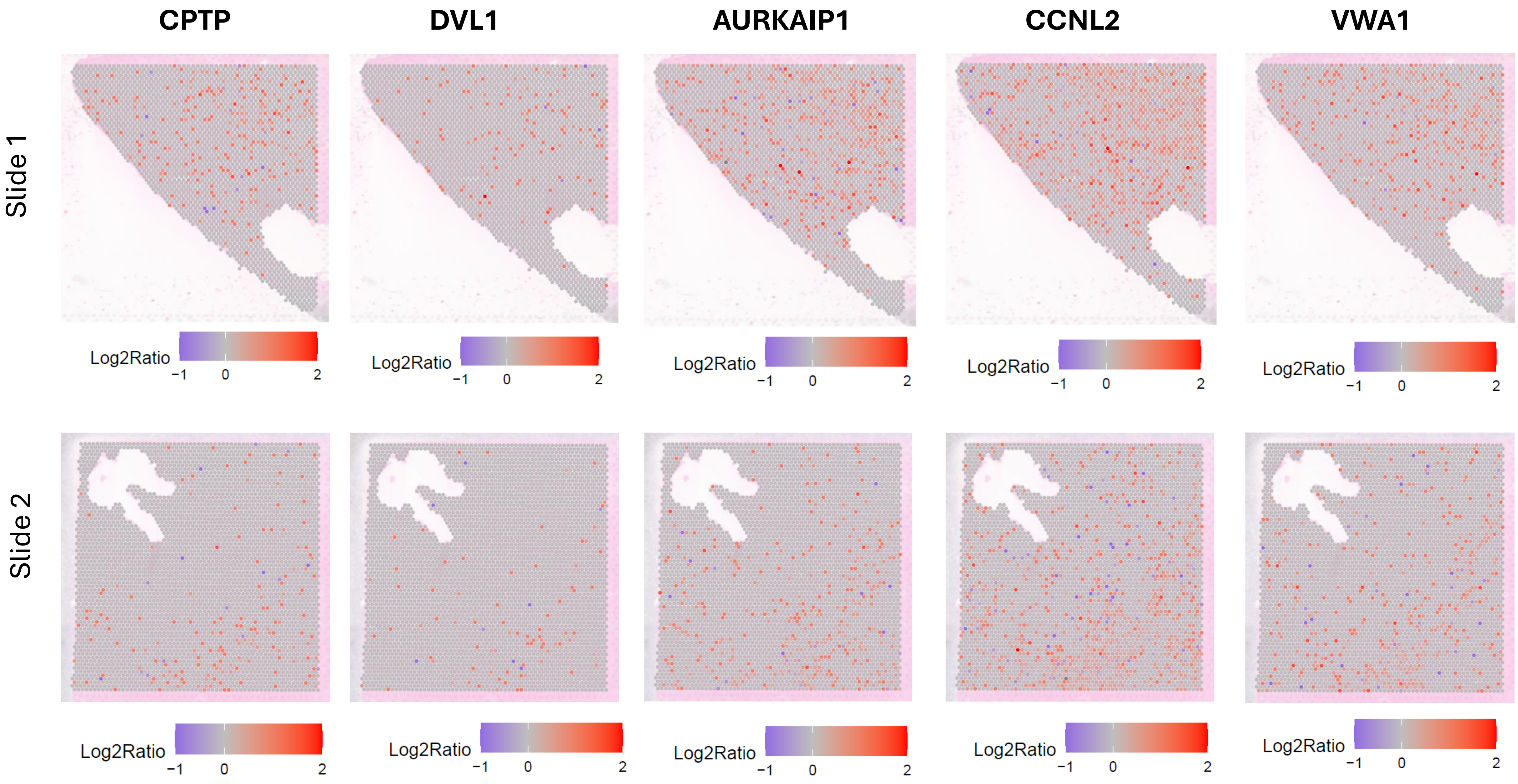

Supplemental figure 7A

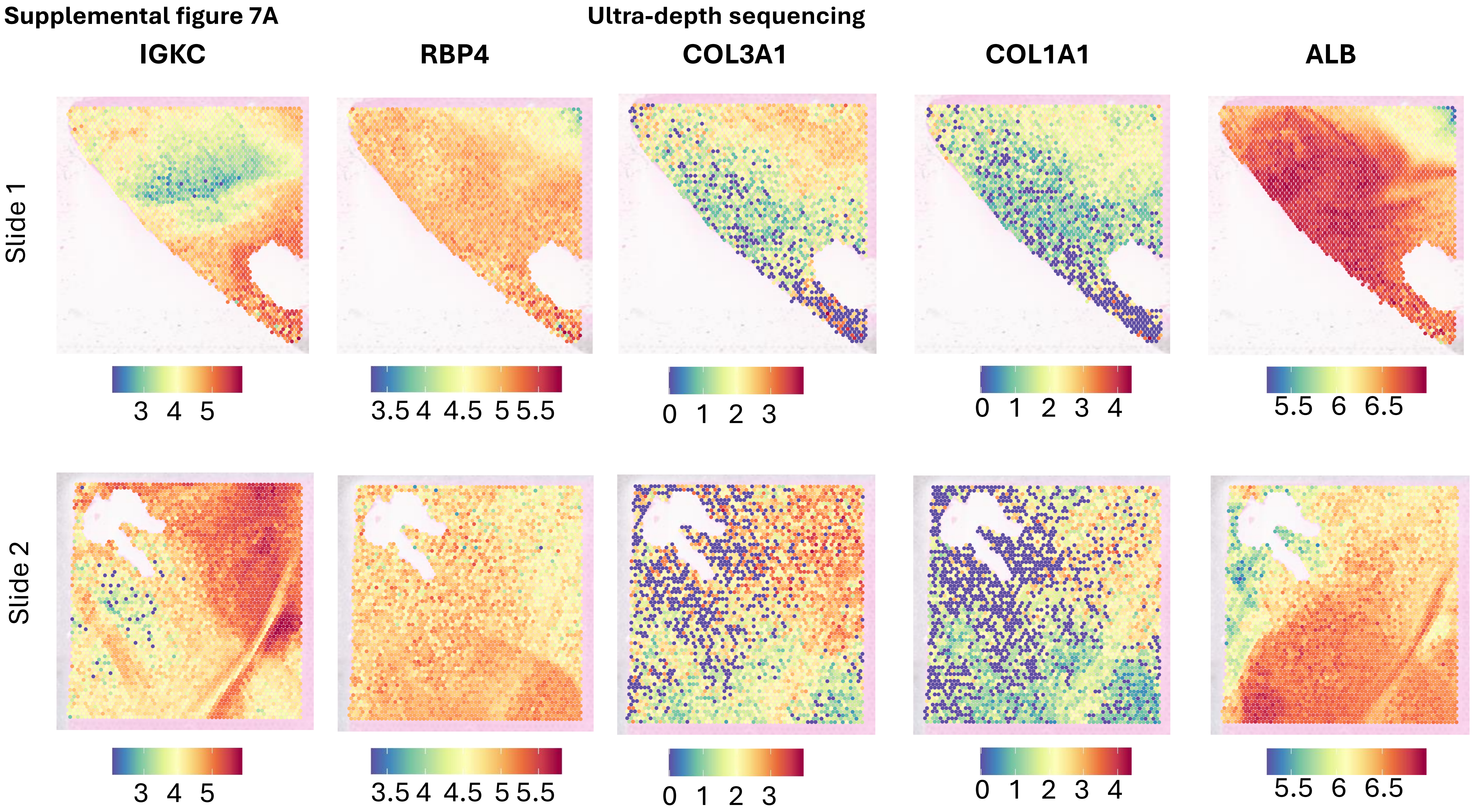

Supplemental figure 7B

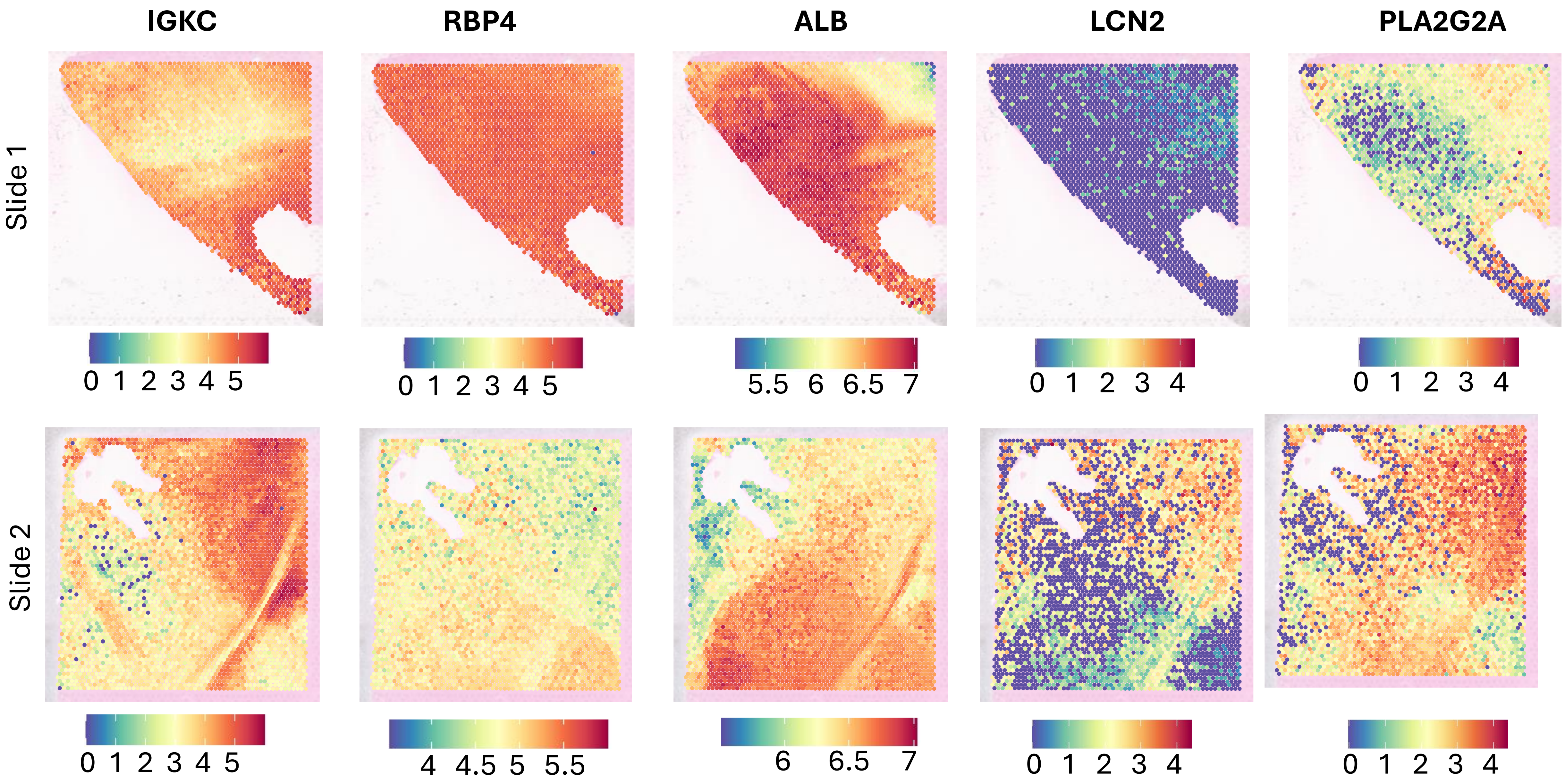

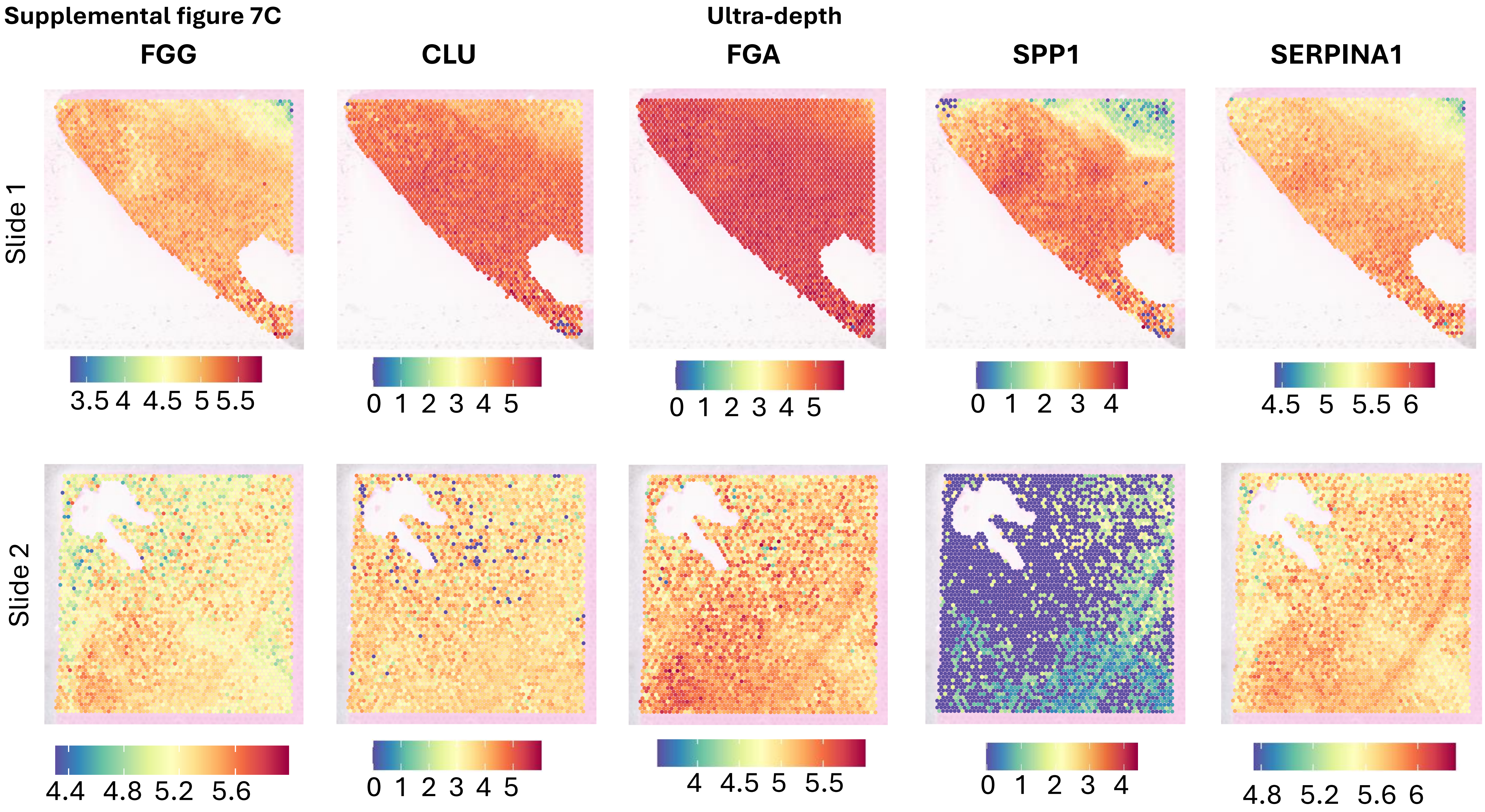
Supplemental figure 7C
Ultra-depth
FGG
CLU
FGA
SPP1
SERPINA1
Slide 1
3.5 4 4.5 5 5.5
0 1 2 3 4 5
0 1 2 3 4 5
0 1 2 3 4
4.5 5 5.5 6
Slide 2
4.4 4.8 5.2 5.6
0 1 2 3 4 5
4 4.5 5 5.5
0 1 2 3 4
4.8 5.2 5.6 6

Supplemental figure 7D

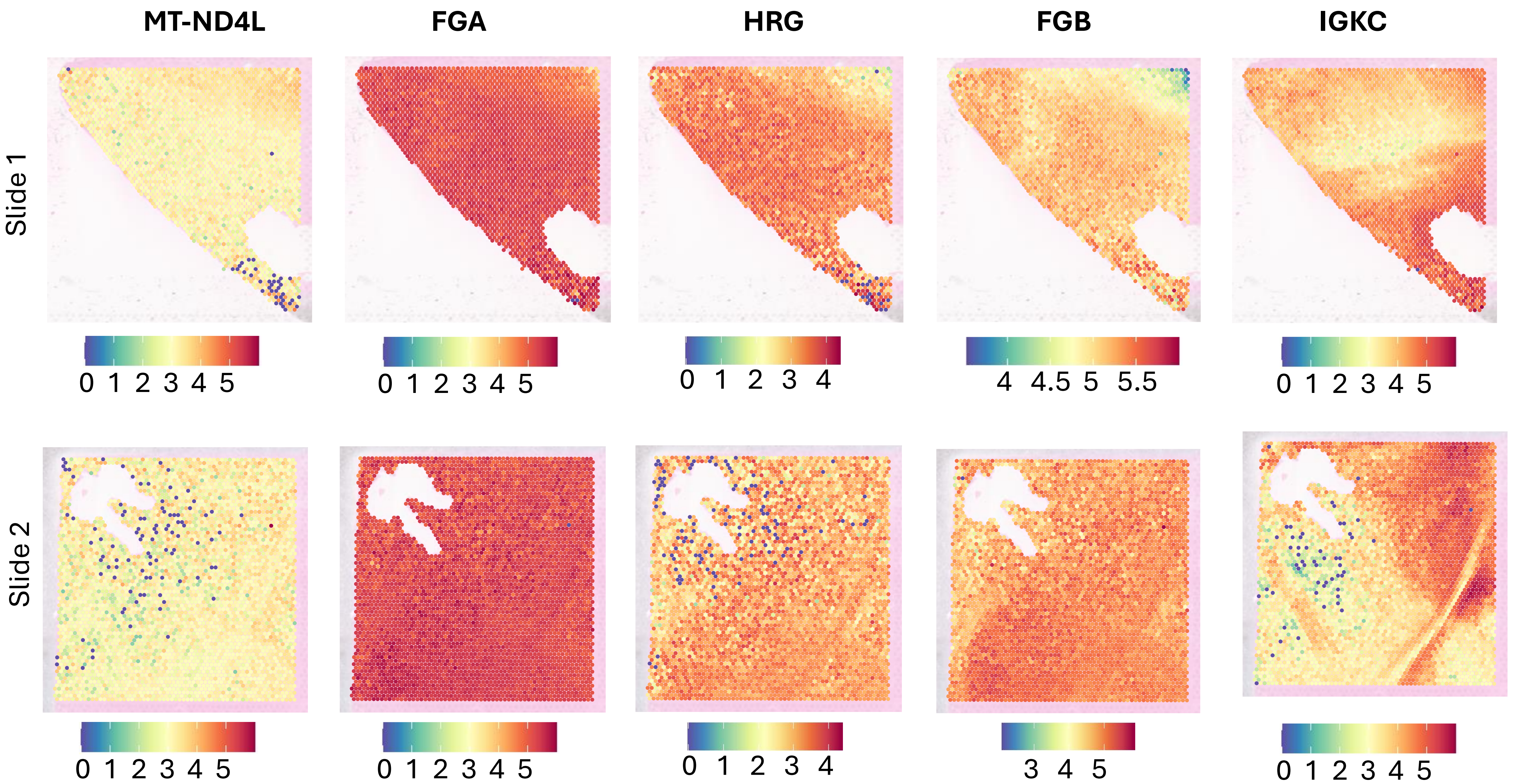

Supplemental figure 8

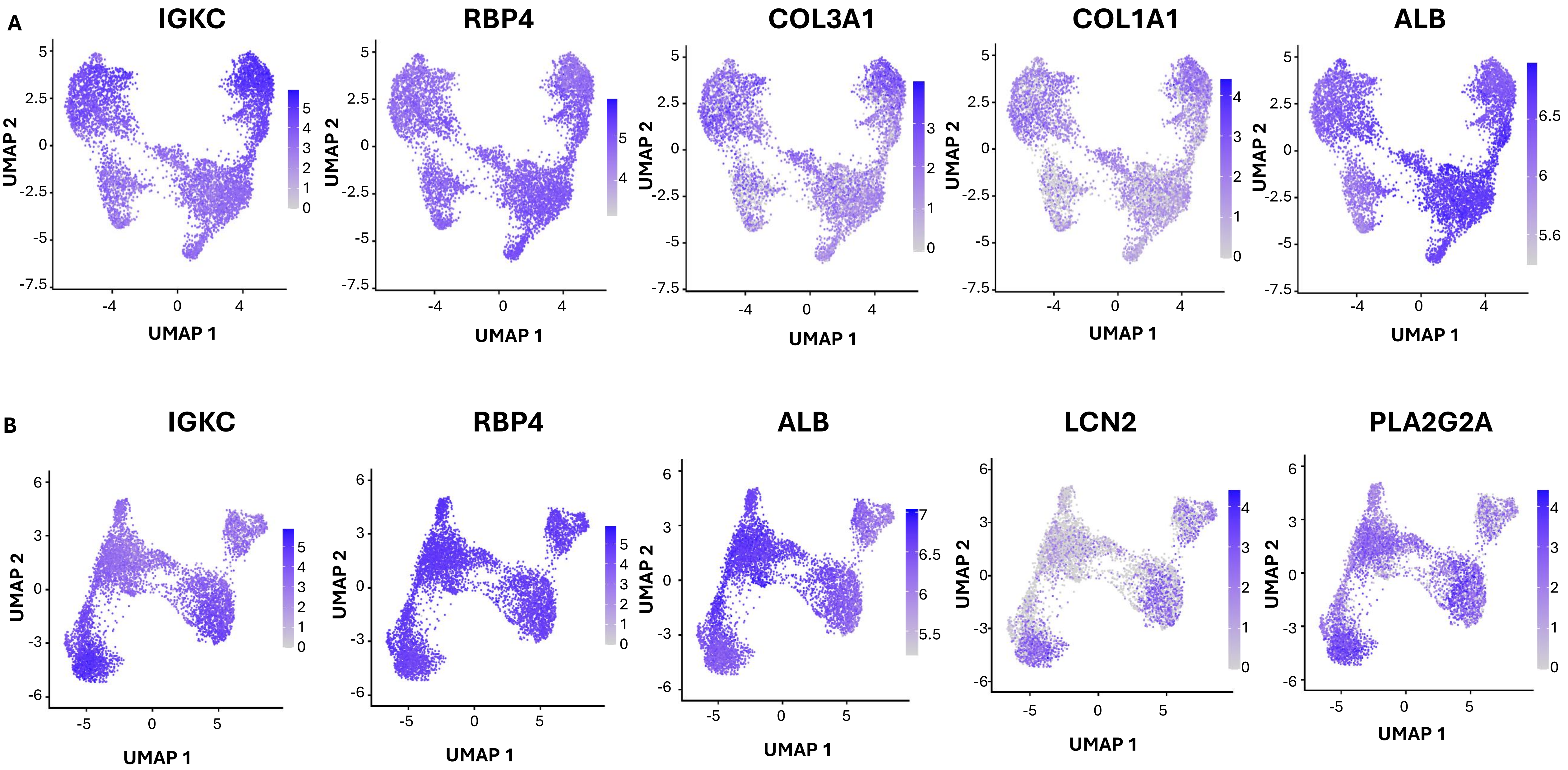

Supplemental figure 8

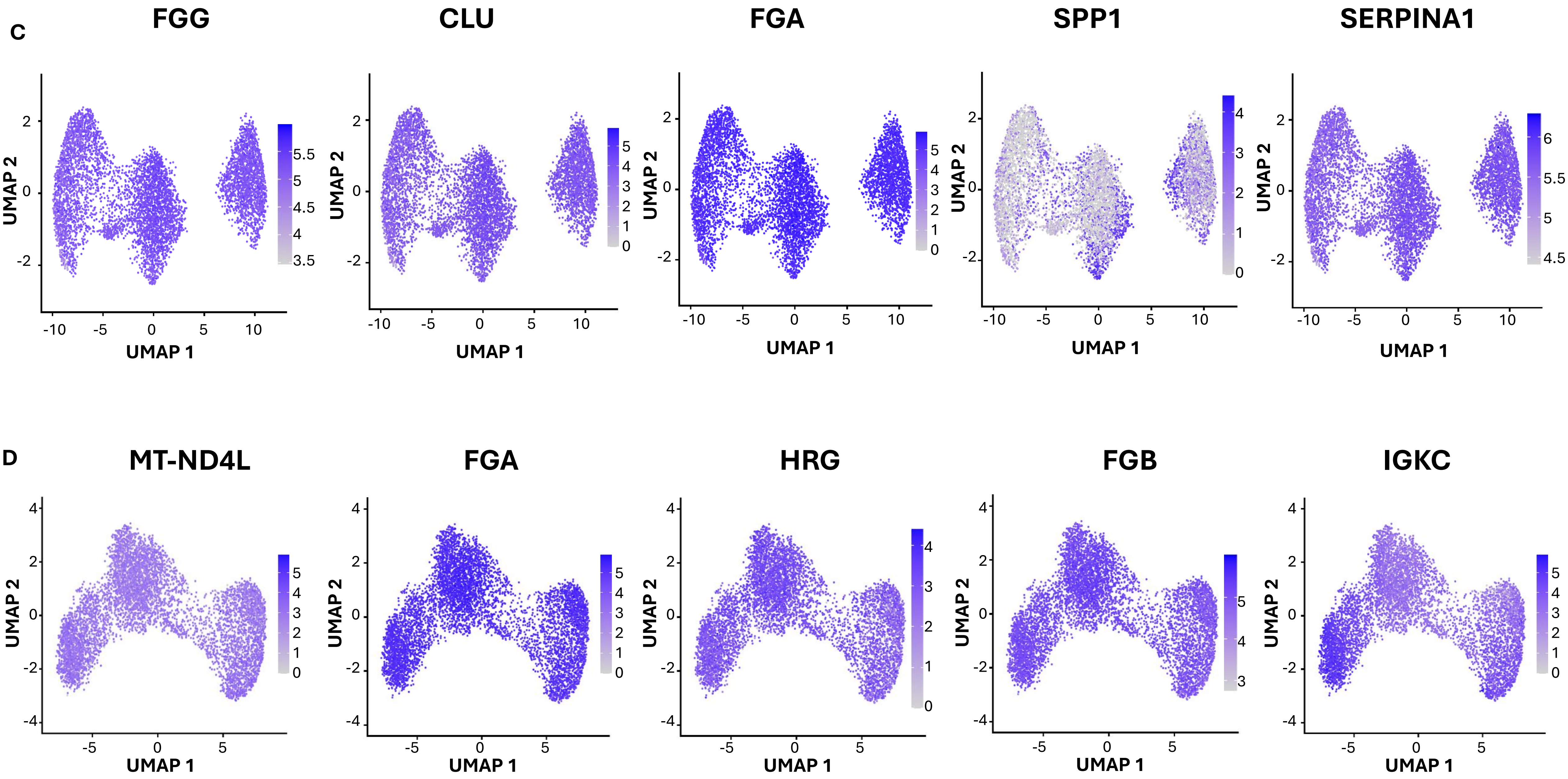

Supplemental figure 9

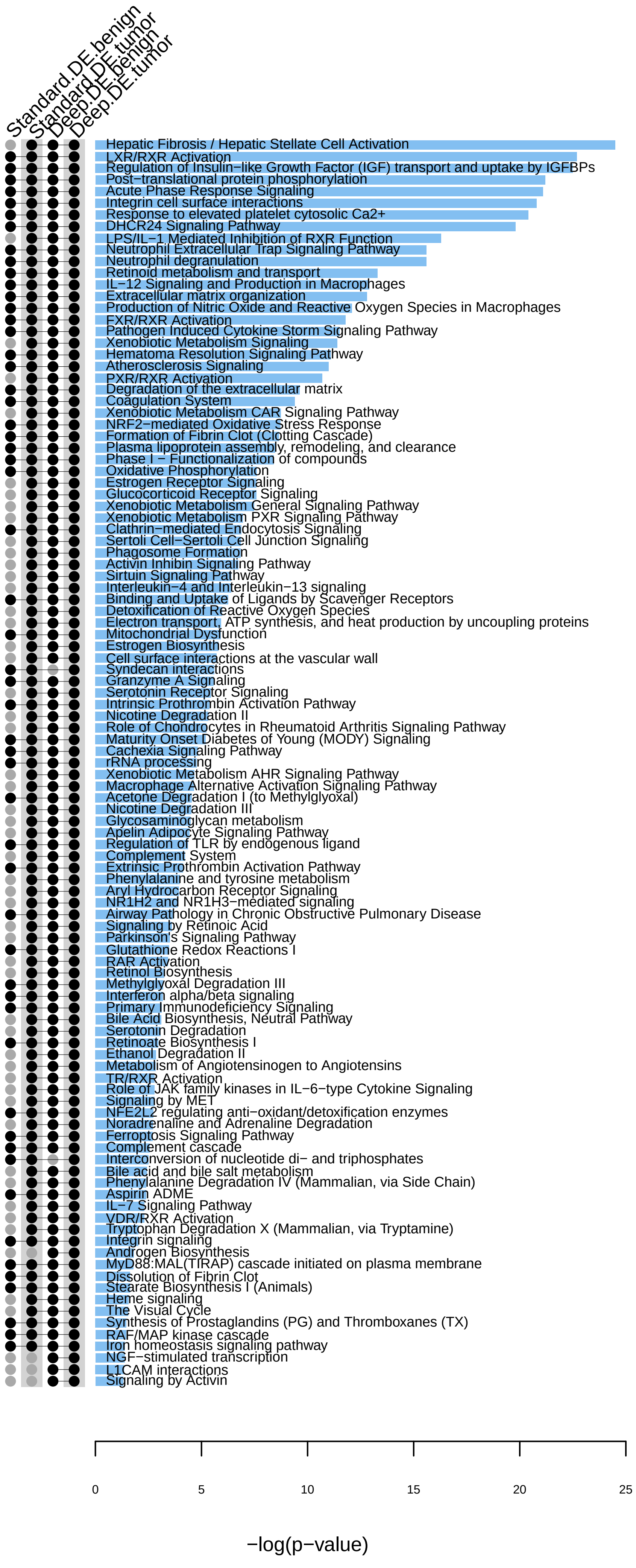

Supplemental figure 10

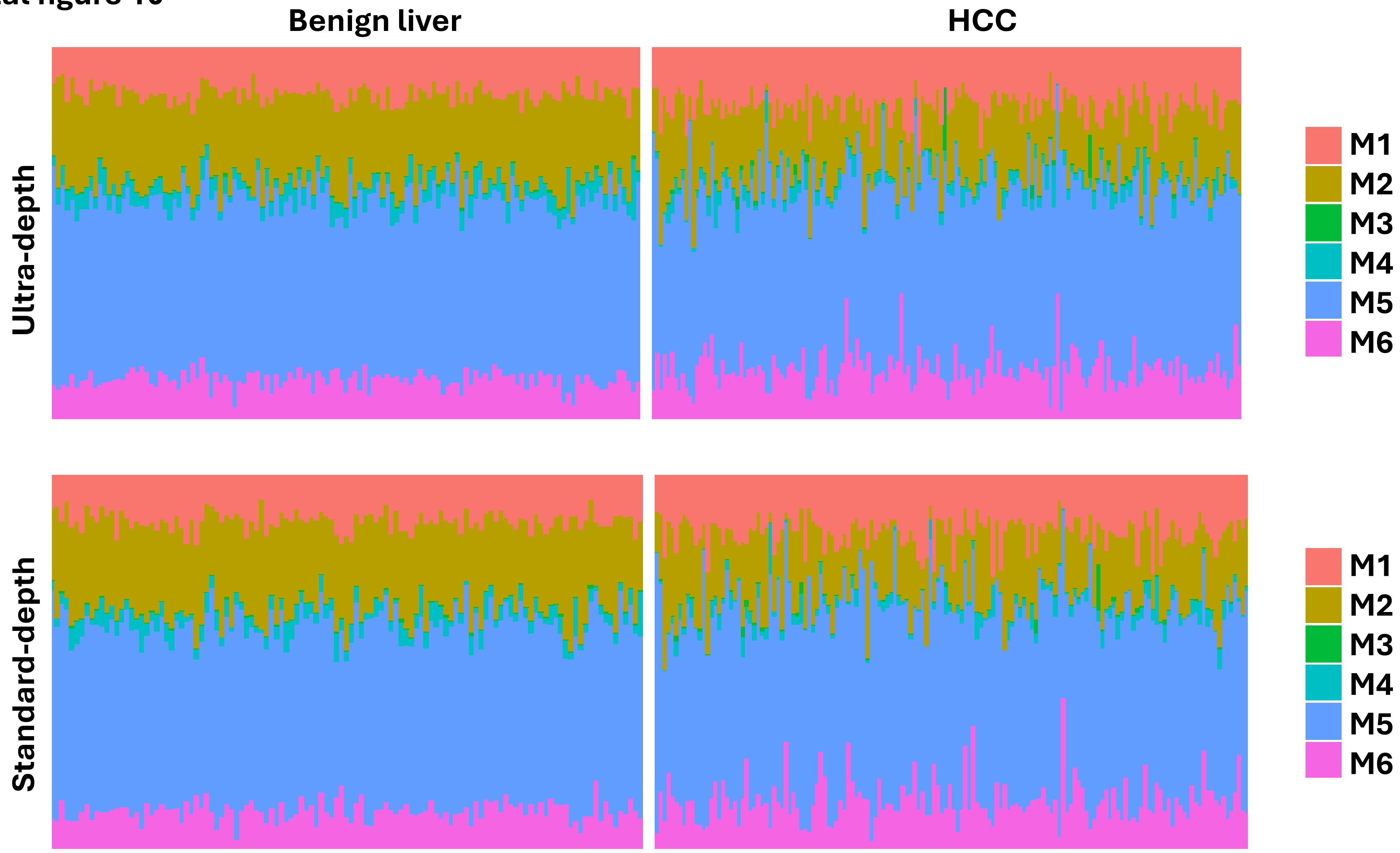

Supplement: Supplementary file 1 [file DataSheet1.pdf]
